# Subtle variation within conserved effector operon gene products contributes to T6SS-mediated killing and immunity

**DOI:** 10.1371/journal.ppat.1006729

**Published:** 2017-11-20

**Authors:** Christopher J. Alteri, Stephanie D. Himpsl, Kevin Zhu, Haley L. Hershey, Ninette Musili, Jessa E. Miller, Harry L. T. Mobley

**Affiliations:** Department of Microbiology and Immunology, University of Michigan Medical School, Ann Arbor, Michigan, United States of America; University of California Davis School of Medicine, UNITED STATES

## Abstract

Type VI secretion systems (T6SS) function to deliver lethal payloads into target cells. Many studies have shown that protection against a single, lethal T6SS effector protein requires a cognate antidote immunity protein, both of which are often encoded together in a two-gene operon. The T6SS and an effector-immunity pair is sufficient for both killing and immunity. HereIn this paper we describe a T6SS effector operon that differs from conventional effector-immunity pairs in that eight genes are necessary for lethal effector function, yet can be countered by a single immunity protein. In this study, we investigated the role that the PefE T6SS immunity protein plays in recognition between two strains harboring nearly identical effector operons. Interestingly, despite containing seven of eight identical effector proteins, the less conserved immunity proteins only provided protection against their native effectors, suggesting that specificity and recognition could be dependent on variation within an immunity protein and one effector gene product. The variable effector gene product, PefD, is encoded upstream from *pefE*, and displays toxic activity that can be countered by PefE independent of T6SS-activity. Interestingly, while the entire *pef* operon was necessary to exert toxic activity via the T6SS in *P*. *mirabilis*, production of PefD and PefE alone was unable to exert this effector activity. Chimeric PefE proteins constructed from two *P*. *mirabilis* strains were used to localize immunity function to three amino acids. A promiscuous immunity protein was created using site-directed mutagenesis to change these residues from one variant to another. These findings support the notion that subtle differences between conserved effectors are sufficient for T6SS-mediated kin discrimination and that PefD requires additional factors to function as a T6SS-dependent effector.

## Author summary

Bacterial type VI secretion systems (T6SS) function as contractile nanomachines to puncture target cells and deliver lethal effectors. Little is known about the lifestyle or physiology dictating when bacteria normally express their T6SS. Previously, we have reported that discrimination of self during the multicellular swarming behavior of *Proteus mirabilis* requires the lethal action of the T6SS, T6SS-dependent effectors, and immunity proteins. Bacteria that share common immunity proteins are protected against T6SS killing and recognize each other as self. Here we introduce a new group of T6SS immunity effectors that require eight genes for killing and a single immunity gene that differs from the classical effector-immunity pairs. Variation within an effector operon drives specificity and recognition between strains. We propose that subtle sequence variation in the effector operons contribute to self and non-self recognition in these bacteria.

## Introduction

The type VI secretion system (T6SS) functions upon cell contact with a target cell to deliver a payload of effectors through a contractile puncturing device [1–4]. This contact-dependent process results in death of a non-immune target cell following the translocation of lethal effectors from the attacker cell cytoplasm into the recipient’s periplasm [5, 6]. Bacteria are also equipped with other mechanisms to eliminate bacterial competitors by a contact-independent method in which bactericidal agents such as bacteriocins and antibiotics are released into the extracellular environment [7]. It is plausible that bacteria with a multicellular lifestyle, which require direct contact and cooperativity, may favor a contact-dependent process to eliminate neighboring competitors [8, 9]. We and others have shown that multicellular behavior exhibited by *Proteus mirabilis* is indeed linked to T6S and thus enables discrimination during cell-cell contact to eliminate non-self bacteria from the population [10, 11]. Based on these findings, we hypothesize that the T6SS must provide an advantage for *P*. *mirabilis* to discriminate, recognize, and kill competitors that would otherwise interfere with or benefit from their cooperative behavior. In support of this, kin discrimination has recently been established in multicellular *Bacillus subtilis* swarming populations as a mechanism to exclude non-kin from colonizing an ecological niche [12].

*P*. *mirabilis* undergoes a unique morphological cycle, differentiating from short (~2 μm in length), rod-like vegetative swimmer cells into hyperflagellated, elongated (~40 μm in length) swarmer cells that allow *Proteus spp*. to swarm rapidly and uniformly in multicellular rafts across a surface, resulting in a bull's-eye pattern on an agar surface. In 1946, Louis Dienes observed that actively swarming strains of *Proteus* have the remarkable ability to form a macroscopic boundary or “Dienes line” at the intersection of opposing bacterial swarms [13, 14]. Formation of the Dienes line occurs only upon direct cell-cell contact between swarms of different *Proteus* isolates, demonstrating the ability of one strain of *Proteus* to distinguish itself from another [13–15]. It is now clear that the Dienes phenomenon is dependent on the T6SS [10]. Lethal action via the T6SS in one of two merging non-identical swarms is sufficient to produce a Dienes demarcation. Only when both isolates have inactive T6SSs do Dienes lines not form in this scenario [10]. These results show that the failure to recognize the opposing cell as self is a result of the lethal action mediated by the effectors delivered by the T6SS. Therefore, immunity against lethal attack equates to recognition and allows for merging of swarm populations [10]. Thus, it is likely that the recipient or target cell is responsible for kin recognition.

The ability of *P*. *mirabilis* strains to recognize each other suggests that there must be a molecular determinant that facilitates kin recognition by providing immunity against lethal attack. It is possible that this recognition determinant is unique to each strain and contributes to the more than 200 Dienes types that have been reported, a diversity that rivals O-serotypes [16]. *P*. *mirabilis* T6SS effector operons are characterized by two genes, *hcp* and *vgrG*, followed by a variable number of additional genes. We speculate that *P*. *mirabilis* strains possess immunity to specific Hcp-VgrG effector operons; thus strains which contain the same immunity and effector proteins will be immune to each other and "self-recognize". Consequently, strains that come into contact with one another that also possess different Hcp-VgrG effector operons results in killing or kin discrimination from the absence of matching sets of effector operons. Similarly, competition in *Vibrio cholerae* occurs between strains with unmatched effector-immunity gene sets [17].

We have shown that *P*. *mirabilis* isolates BB2000 and HI4320 are capable of killing each other [10] and that non-self recognition by these strains is dependent on differences in their T6SS effector operons [10, 11]. For example, adjacent to the T6SS operon, strain BB2000 contains the *idr* locus that has been shown to play a role in identity recognition [11], while in HI4320 the *pef* effectors are encoded next to the T6SS encoding genes [10]. Lack of immunity between HI4320 and BB2000 could be explained due to the absence of the *idr* locus in HI4320 and the lack of the *pef* operon in BB2000, or both. However, T6SS-dependent kin recognition likely involves additional effectors; for example, we have shown the presence of four distinct orphan Hcp-VgrG effector operons in HI4320 [10], three of which are absent in BB2000. These differences likely also play a role in kin discrimination via the T6SS.

Variation within contact-dependent inhibition (CDI) toxin and immunity pairs has been shown to affect kin recognition [18, 19]. Thus, even when two isolates possess the same complement of Hcp-VgrG effector operons, small sequence variations within these operons could drive T6SS immunity, and therefore recognition differences. For example, variable sequences have been noted for the orphan *ids hcp-vgrG* operon between strains BB2000 and HI4320 [20]. Despite the presence of the *ids hcp-vgrG* effector operon in both strains, the ability of HI4320 and BB2000 to kill one another may suggest that slight variation within the *ids* operon can result in different immunity functions [20, 21]. We propose that it is both the presence of different Hcp-VgrG and effector gene products and sequence variation within matching *hcp-vgrG* effector operons among strains that cumulatively result in two different isolates killing each other. A multi-factorial approach to establishing relatedness in multicellular bacteria could amplify the selectivity of kin/non-kin recognition [22].

In this study, we provide evidence that variation in a conserved effector operon contributes to strain diversity and social recognition in *P*. *mirabilis*. Previously, we have shown that the immunity protein PefE in HI4320 is necessary and sufficient for immunity against HI4320 attack and is necessary but not sufficient for killing [10]. We now show that by interchanging effector-immunity gene variants within the same effector operon, we can affect kin recognition. Additionally, independent of T6SS activity, we found that the PefE immunity protein can protect bacterial cells from toxicity of a predicted nuclease effector, PefD. However, unlike introduction of the entire *pef* operon into T6SS-active *P*. *mirabilis*, the introduction of a *pefDE* pair was not sufficient to provide a new means to discriminate non-identical swarms or produce a new Dienes type indicating that specificity requires additional factors.

## Results

### *P*. *mirabilis* encodes T6SS effector-immunity pairs within larger operons

**Fig 1:**
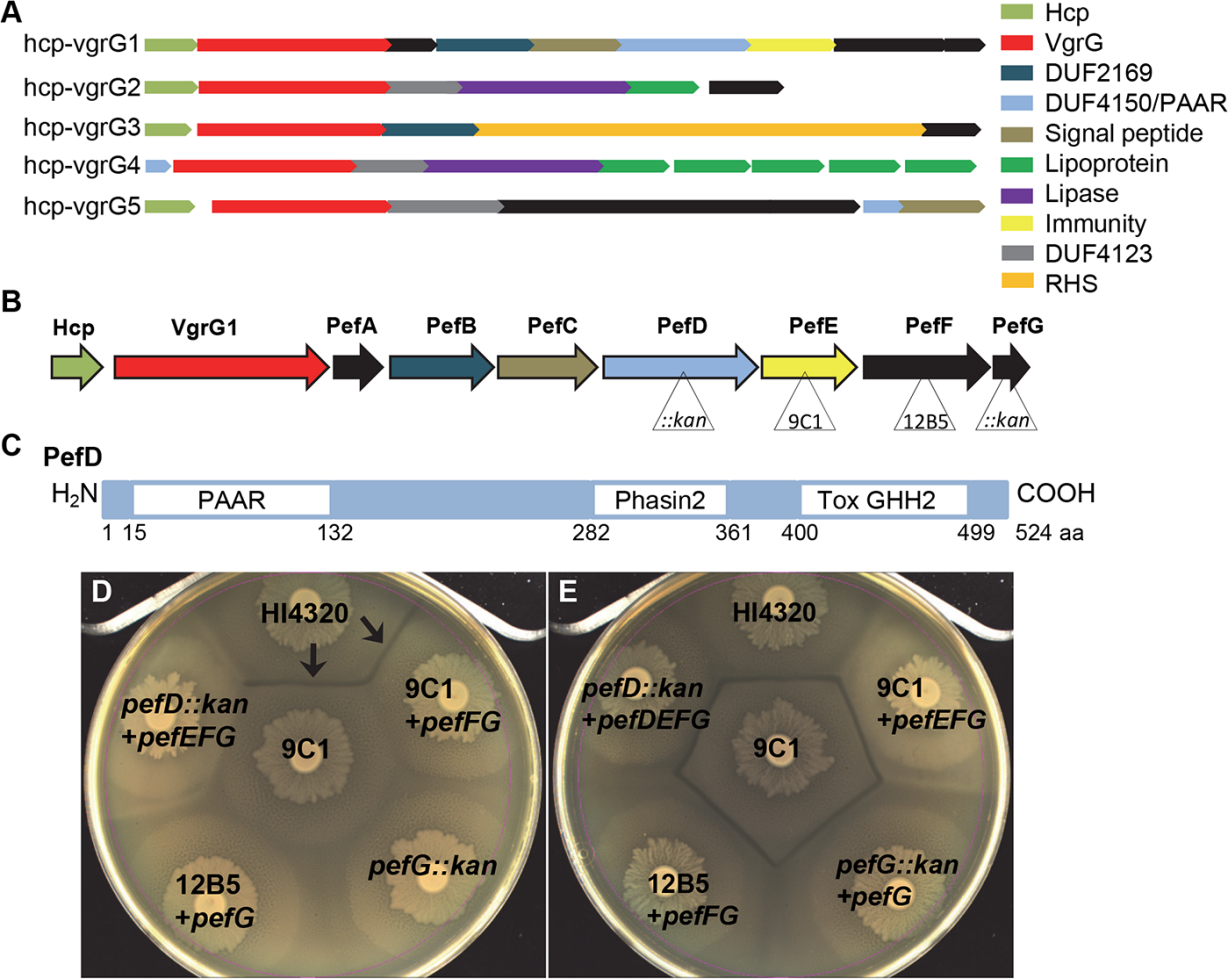
Organization of and requirement for multiple genes within T6SS effector operons in *P*. *mirabilis*. **(A)** T6SS effector operons in *P*. *mirabilis* HI4320 all include *hcp*, *vgrG*, and between five and nine additional genes. **(B)** Organization of the *hcp-vgrG1* primary effector (*pef)* operon in *P*. *mirabilis* HI4320. Position of insertion mutations are indicated by triangles. **(C)** Schematic of the predicted nuclease toxin PefD. The position of the conserved domains are indicated; PAAR, Phasin2, and Tox GHH2. **(D)** Parental wild-type *P*. *mirabilis* HI4320 forms a Dienes line with *pefE* mutant bacteria (black arrows) and mutants lacking single genes *pefD*, *pefE* (9C1), *pefF* (12B5), or *pefG* each fail to form the boundary with the immunity mutant (center). **(E)** Complementation restores T6SS-dependent boundary formation, indicating the requirement for multiple T6SS genes for effector function. In (A) and (B) predicted conserved domains are color-coded and include DUF2169, DUF4150, DUF4123, PAAR-containing domains, lipoprotein, lipase, and RHS domains. The known immunity-encoding gene *pefE* is in yellow.

**Fig 2:**
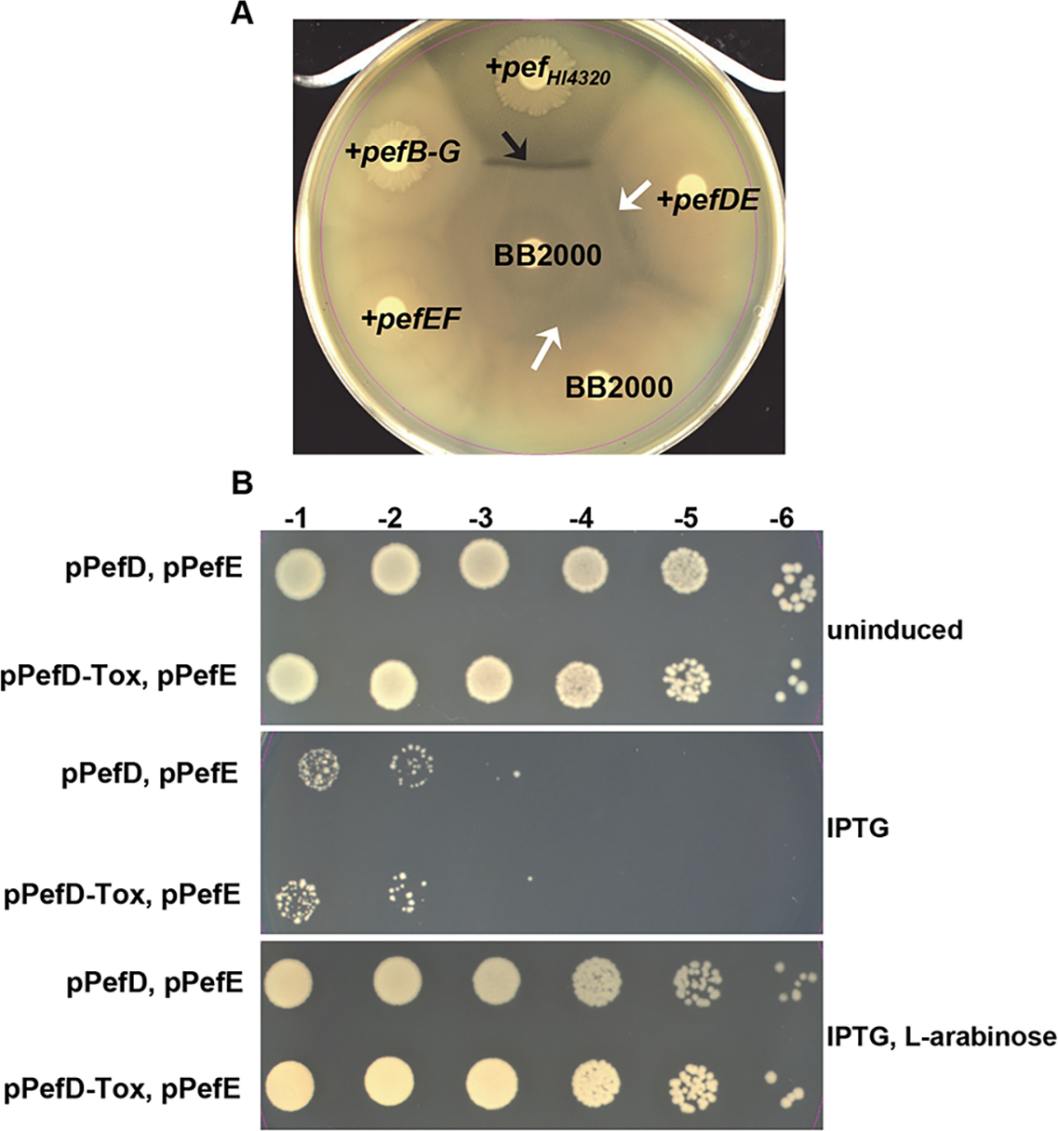
PefD and PefE can function as a toxin/anti-toxin independent of T6SS activity. **(A)** Introduction of the entire *pef* operon from strain HI4320 into *P*. *mirabilis* BB2000 is sufficient to form a Dienes line with wild-type parental strain BB2000 (black arrow), while PefD and PefE alone are not sufficient for T6SS-dependent effector activity in *P*. *mirabilis* BB2000 and produce a result identical to the lack of a boundary between isogenic swarms of wild-type BB2000 (white arrows). **(B)** Serial dilutions of *E*. *coli* containing PefD and PefE, or the predicted nuclease domain of PefD (PefD-Tox) and PefE, on LB agar (uninduced), agar containing IPTG to induce PefD or PefD-Tox, and agar containing IPTG and L-arabinose to additionally induce PefE. Toxicity is observed when PefD or PefD-Tox are induced in the absence of PefE induction.

Previously, we have shown that the *P*. *mirabilis* HI4320 genome contains a 16-gene operon encoding the structural components of the T6SS and five additional operons of 5–9 genes that begin with *hcp* and *vgrG* [10]. We reasoned that the five *hcp-vgrG* operons encode distinct effector and immunity functions that can be deployed by the T6SS. Interestingly, none of the operons are organized as effector-immunity pairs (Fig 1A). Since the majority of bacteria with characterized T6SSs encode their effectors in two-gene operons, consisting of a toxin and immunity gene product, we first determined if an effector and immunity pair could readily be identified within the *P*. *mirabilis pef* operon (Fig 1B). Indeed, the *pef* operon encodes a protein (PefD) that has a PAAR domain at the amino terminus and a predicted HNH nuclease domain at the carboxy terminus (Fig 1C). The *pefD* gene is found immediately upstream from *pefE*, which we previously demonstrated is required for immunity [10]. This arrangement is consistent with a two gene T6SS effector-immunity pair within the larger *pef* operon context. We next wanted to assess whether or not an entire effector operon is required for function. To achieve this, we used both loss-of-function and gain-of-function approaches. We found that mutants in HI4320 lacking *pefD* were unable to form a Dienes line with the immune-deficient *pefE* mutant (Fig 1D), suggesting that *pefD* is required for effector activity. However, we also found that *pefE*, *pefF*, and *pefG* were also required to form the Dienes line with the strain lacking *pefE* (Fig 1D). We have previously noted that these mutations likely have polar effects and to gain further insight into individual gene product functions we tested complementation with either the mutated gene plus the downstream genes from the *pef* operon, or with only the downstream genes (Table 1). Complementation with only the genes downstream from the mutation did not affect the results (Fig 1D); however, complementation of the downstream genes with the restored mutated gene fully complements the ability to form a Dienes line with the immunity mutant (Fig 1E). We also assessed whether introduction of the genes encoding the putative PefDE effector-immunity pair would function in another T6SS-proficient strain, BB2000, which lacks any *pef* genes in its genome [23]. As expected, based on the mutant phenotypes, *pefDE* alone were not sufficient to produce a Dienes line (Fig 2A, white arrows), unlike the introduction of the entire HI4320 *pef* operon into BB2000 (Fig 2A, black arrow). These data do not rule out the possibility of an effector-immunity pair within the *pef* operon but does suggest that *P*. *mirabilis* T6SS *pef-*encoded effector function requires additional genes.

**Table 1:** Strains and plasmids.

**Strain**	**Description**	**Reference**
***P*. *mirabilis* HI4320**	**clinical isolate**	**[31]**
**HI4320 9C1**	**Tn5 insertion in *pefE***	**[10, 33]**
**HI4320 *pefD*::*kan***	**TargeTron mutation in *pefD***	**[10]**
**HI4320 12B5**	**Tn5 insertion in *pefF***	**[10, 33]**
**HI4320 *pefG*::*kan***	**TargeTron mutation in *pefG***	**[10]**
***P*. *mirabilis* BB2000**	**clinical isolate**	**[34]**
***E*. *coli* BL21 DE3 pLysS**	**protein expression strain**	
***P*. *mirabilis* BA6163**	**clinical isolate**	**[32]**
***P*. *mirabilis* HU1069**	**clinical isolate**	**[32]**
**Plasmid**	**Description**	**Reference**
**pBAD-MycHisA**	**cloning vector**	
**pGEN-MCS**	**cloning vector**	**[35]**
**pBAD*pefEFG***	***pefEFG* from HI4320**	**[10]**
**pBAD*pefDEFG***	***pefDEFG* from HI4320**	**This work**
**pBAD*pefFG***	***pefFG* from HI4320**	**[10]**
**pBAD*pefG***	***pefG* from HI4320**	**[10]**
**pBAD*pefDE***	***pefDE* from HI4320**	**This work**
**pBAD*pefEF***	***pefEF* from HI4320**	**[10]**
**pBAD*pefBCDEFG***	***pefBCDEFG* from HI4320**	**This work**
**pBAD*pefE***	***pefE* from HI4320**	**[10]**
**pGEN*pefHI***	***pef* operon from HI4320**	**[10]**
**pGEN*pefBA***	***pef* operon from BA6163**	**This work**
**pBAD*pefE2***	***pefE2* from HI4320**	**This work**
**pBAD*pefE* HU1069**	***pefE* from HU1069**	**This work**
**pETite N-HIS-PefD**	**PefD from HI4320**	**This work**
**pETite N-HIS-Tox-PefD**	**PefD Tox GHH2 from HI4320**	**This work**

### PefD alone is toxic in *E*. *coli* and PefE protects from the toxicity of PefD

While PefD appears insufficient to promote native T6SS effector activity in *P*. *mirabilis*, we tested for possible PefD toxic activity by overexpressing the protein in *E*. *coli*. Sequences encoding Holo-PefD and a PefD containing only the C-terminal putative nuclease domain (PefD-Tox) were cloned and expressed in *E*. *coli* under the control of an IPTG-inducible promoter. When induced with IPTG, both PefD and PefD-Tox exhibited growth inhibition in *E*. *coli* (Fig 2B). This toxic phenotype was not observed in uninduced bacteria or when PefE was also co-expressed under an arabinose-inducible promoter (Fig 2B). The growth inhibition by PefD and PefD-Tox was also observed during culture in liquid medium only in the presence of IPTG induction (S1 Fig), however, in these conditions PefE was not able to reverse the growth inhibition (S2 Fig). These data support the notion that PefD is a toxic effector and PefE can act as an immunity factor in the absence of T6SS activity in *E*. *coli* (Fig 2B).

### *P*. *mirabilis* HI4320 encodes two functionally distinct PefE immunity proteins

**Fig 3:**
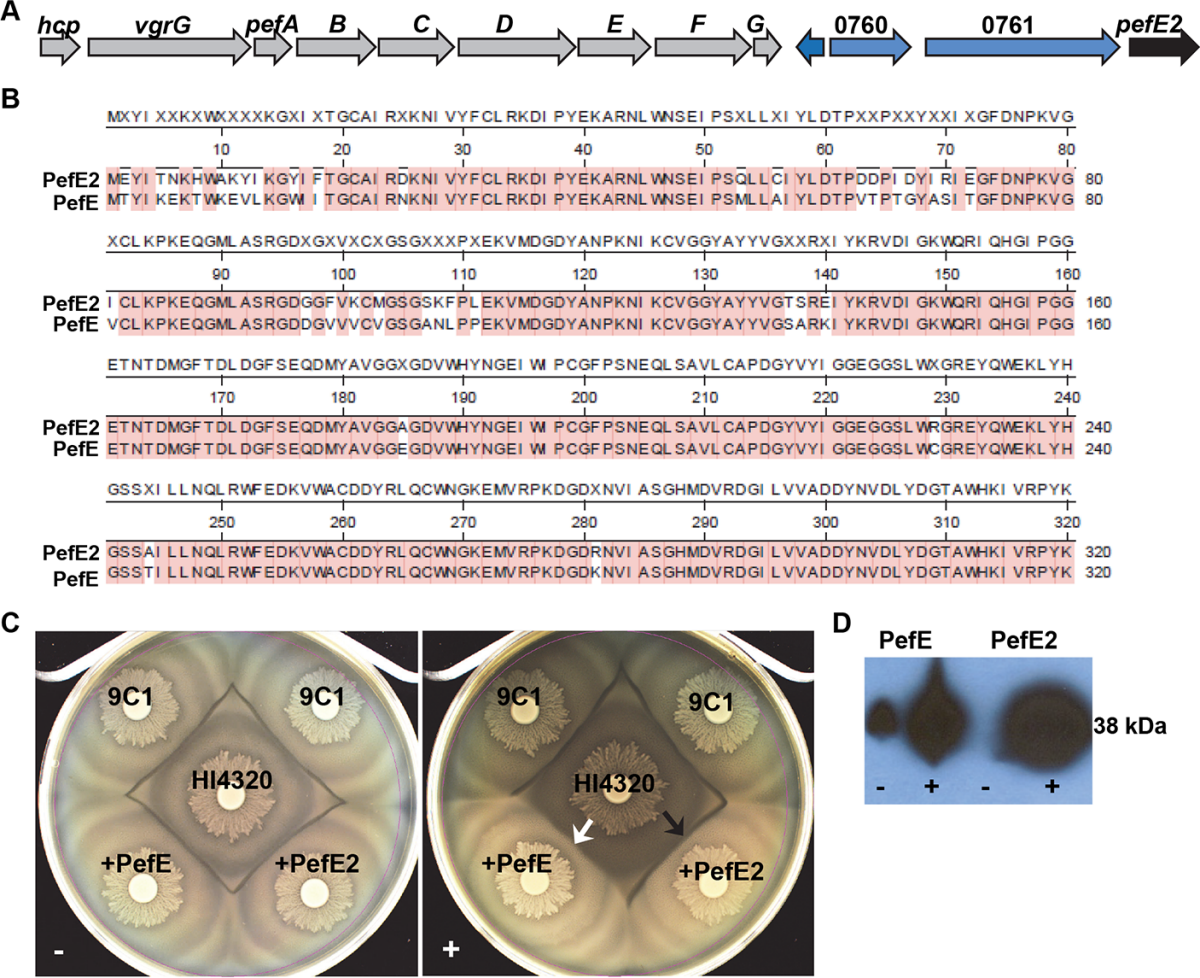
*P*. *mirabilis* HI4320 encodes two functionally distinct PefE proteins. **(A)** The primary effector operon; *hcp*, *vgrG*, *pefABCDEFG*, in HI4320 (grey arrows). Downstream genes from the *pef* operon include a second copy of *pefE (pefE2*, black arrow) located next to two transposase pseudogenes (PMI0759 and PMI0760) and a recombination hot-spot (*rhs*) gene fragment (PMI0761) depicted as blue arrows. **(B)** Alignment of HI4320 PefE and PefE2 predicted amino acid sequences. Amino acids shaded in red indicate residue similarities (284/320 amino acids, 89% identity) between HI4320 PefE and PefE2. **(C)** Dienes line formation is observed under uninduced conditions (-) with 9C1 containing empty vector, 9C1 containing *pefE*, and 9C1 containing *pefE2* against parent strain, HI4320. Following induction on 10 mM L-arabinose (+), expression of *pefE2* in 9C1 does not complement the immunity defect caused by disruption of *pefE* (black arrow) against parent strain HI4320. Only *pefE* expressed in 9C1 restores immunity (white arrow) against HI4320. **(D)** Western blot of 6xHIS tagged PefE and 6xHIS tagged PefE2 expressed during growth in LB broth with (+) and without (-) 10 mM L-arabinose.

We noted that the *P*. *mirabilis* HI4320 genome encodes an additional copy of *pefE* (*pefE2;* PMI0762) a few genes downstream of the *pef* operon (Fig 3A). Located immediately upstream of *pefE2* are two transposase pseudogenes and a recombination hot-spot (*rhs*) gene fragment encoded by PMI0759, PMI0760 and PMI0761, respectively (Fig 3A). Alignment of amino acid sequences of, PefE and PefE2, showed that the highly conserved proteins share 284/320 amino acid residues (89% identity) (Fig 3B). The majority of these differences are located in the N-terminal half of PefE (Fig 3B). The high conservation between *pefE* and *pefE2* was surprising because loss of *pefE* in HI4320 (strain 9C1) results in a kin recognition defect, where parental HI4320 forms a Dienes line with 9C1 (Fig 3C). Two possibilities may explain this result. Either *pefE2* is not functionally equivalent to *pefE* or *pefE2* is not expressed. To test these possibilities, we cloned *pefE2* with a C-terminal epitope tag under an arabinose-inducible promoter to force expression in the 9C1 mutant; however, *pefE2* could not complement the *pefE* mutation and a Dienes line formed (Fig 3C, black arrow). To confirm production of PefE2, the bacteria with the construct were cultured in LB medium with and without 10 mM L-arabinose and a western blot was performed with a 6XHis antibody that binds to the PefE2 epitope tag (Fig 3D). Visible expression of PefE2-6XHis by the 6XHis antibody rules out lack of expression as a reason for the inability of PefE2 to restore immunity in the 9C1 mutant and suggests that one or more of the amino acid residue differences in the N-terminus region of PefE and PefE2 result in functional differences. These findings suggest that the protein encoded by *pefE* provides immunity to the *pef* locus while the second copy, *pefE2*, does not appear to play a role in immunity to the *pef* effector operon gene products from HI4320 and thus, may be an orphan immunity factor.

### Sequence variation of immunity proteins create functionally distinct effector operons

**Fig 4:**
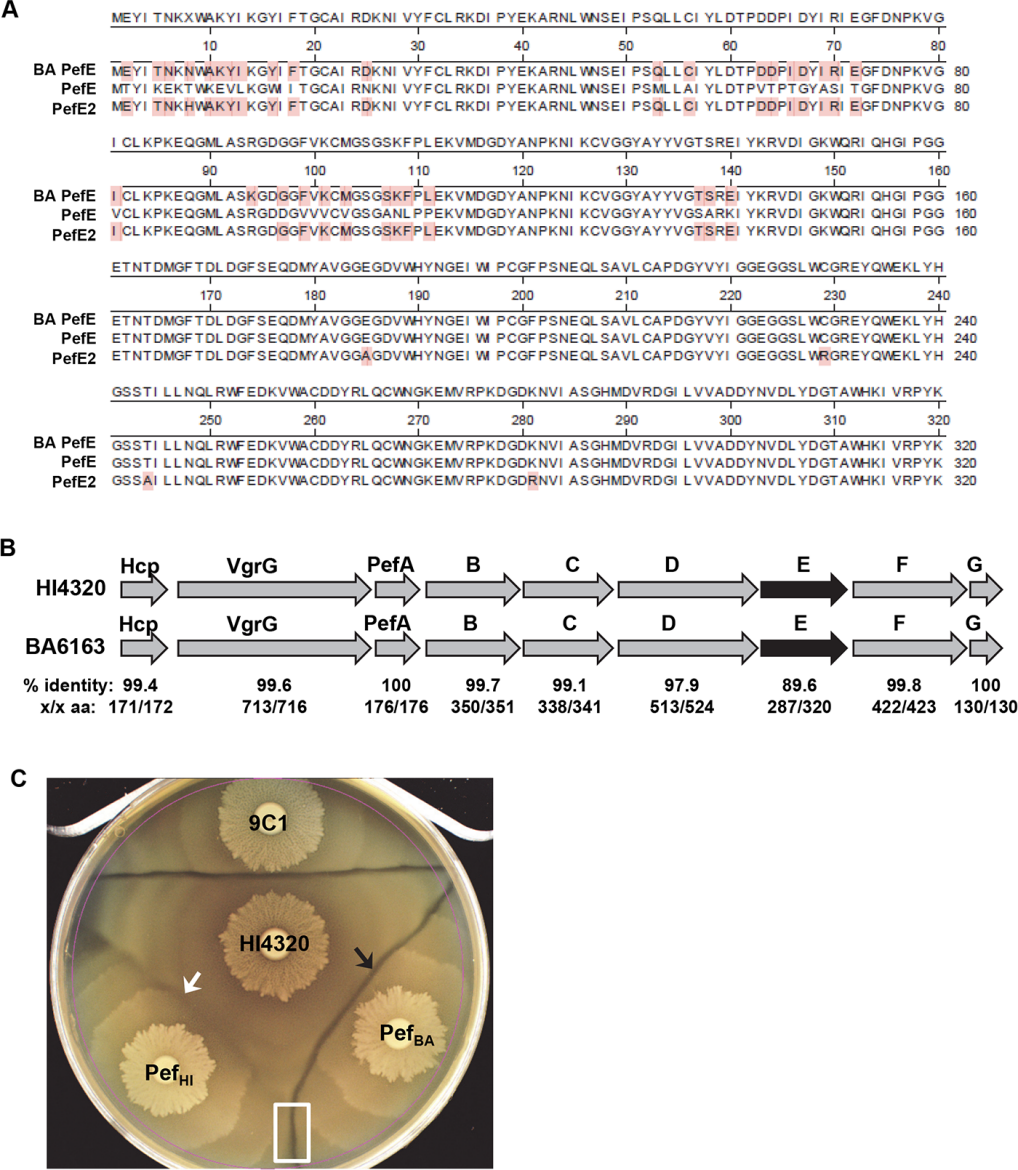
Comparison of protein sequences of PefE2 of *P*. *mirabilis* HI4320 and PefE of BA6163 shows high level of conservation. **(A)** Amino acid sequence alignments indicate that PefE of BA6163 is more similar to PefE2 than PefE of HI4320 with 315/320 shared amino acids (98% identity). Amino acids shaded in red indicate residue similarities between BA6163 PefE and HI4320 PefE2 that differ from PefE of HI4320. **(B)** Arrows represent the gene products of the primary effector operon in HI4320 and BA6163. Percent identity of the amino acid sequences comparison and number of conserved amino acids are listed for each protein. PefE (shaded black) has the lowest percent identity, 89.6%, of the two strains. **(C)** Following induction on 10 mM L-arabinose, 9C1 containing the BA6163 *pef* operon (Pef_BA_) was unable to complement 9C1 immunity resulting in a visible Dienes line (black arrow) with parent strain, HI4320, while 9C1 containing the HI4320 *pef* operon (Pef_HI_) complements immunity against HI4320 (white arrow). The boxed area in (C) indicates that 9C1 containing Pef_HI_ and Pef_BA_ are restored for killing and are not immune to one another.

In our previous study, we identified another *P*. *mirabilis* strain, BA6163, that contained sequence homology to a small region of *pefE* [10]. Sequencing of the BA6163 *pefE* gene revealed that BA6163 encodes a full length *pefE*; however, the deduced amino acid sequence alignment indicates that the BA6163 PefE is more similar to HI4320 PefE2 than PefE (Fig 4A). Specifically, PefE of BA6163 and PefE2 of HI4320 share 315/320 amino acids (98% identity) while PefE of BA6163 and PefE of HI4320 only share 287/320 amino acids (89.6% identity) (Fig 4A). To determine if the BA6163 *pefE* is an “orphan” like *pefE2* in HI4320 or part of an effector operon like *pefE*, we cloned and sequenced the entire *pef* region of BA6163. The amino acid sequence of proteins encoded by the *pef* operon from BA6163 are remarkably similar to the proteins encoded by the *pef* operon from HI4320 (97.9% to 100% identity) with the major differences located in PefE (Fig 4B). Seven of the nine proteins; Hcp, VgrG, PefA, PefB, PefC, PefF, and PefG were all greater than 99% identical between BA6163 and HI4320 (Fig 4B). PefD is also highly conserved, 97.9% identical (513/524 residues), while PefE is least conserved, 89.6% identical (287/320residues), between BA6163 and HI4320 (Fig 4B). Since *pefE2* did not complement 9C1 (Fig 3C), we predicted that the entire *pef* operon from BA6163 would also fail to complement 9C1. Indeed, the BA6163 *pef* operon was unable to complement the HI4320 9C1 *pefE* mutation (Fig 4C), while introduction of the HI4320 *pef* operon does complement 9C1 (Fig 4C). Interestingly, a Dienes line (boxed area) was observed between the 9C1 strains containing the BA6163 *pef* operon and the *pef* operon from HI4320 (Fig 4C). These findings suggest that the *pef* operon of BA6163 and the *pef* operon of HI4320 are functionally different and have unique specificities based upon their divergent PefE immunity proteins, variability within the PefD protein, or both.

### The *pef* operon is sufficient to create new Dienes-types in *P*. *mirabilis*

**Fig 5:**
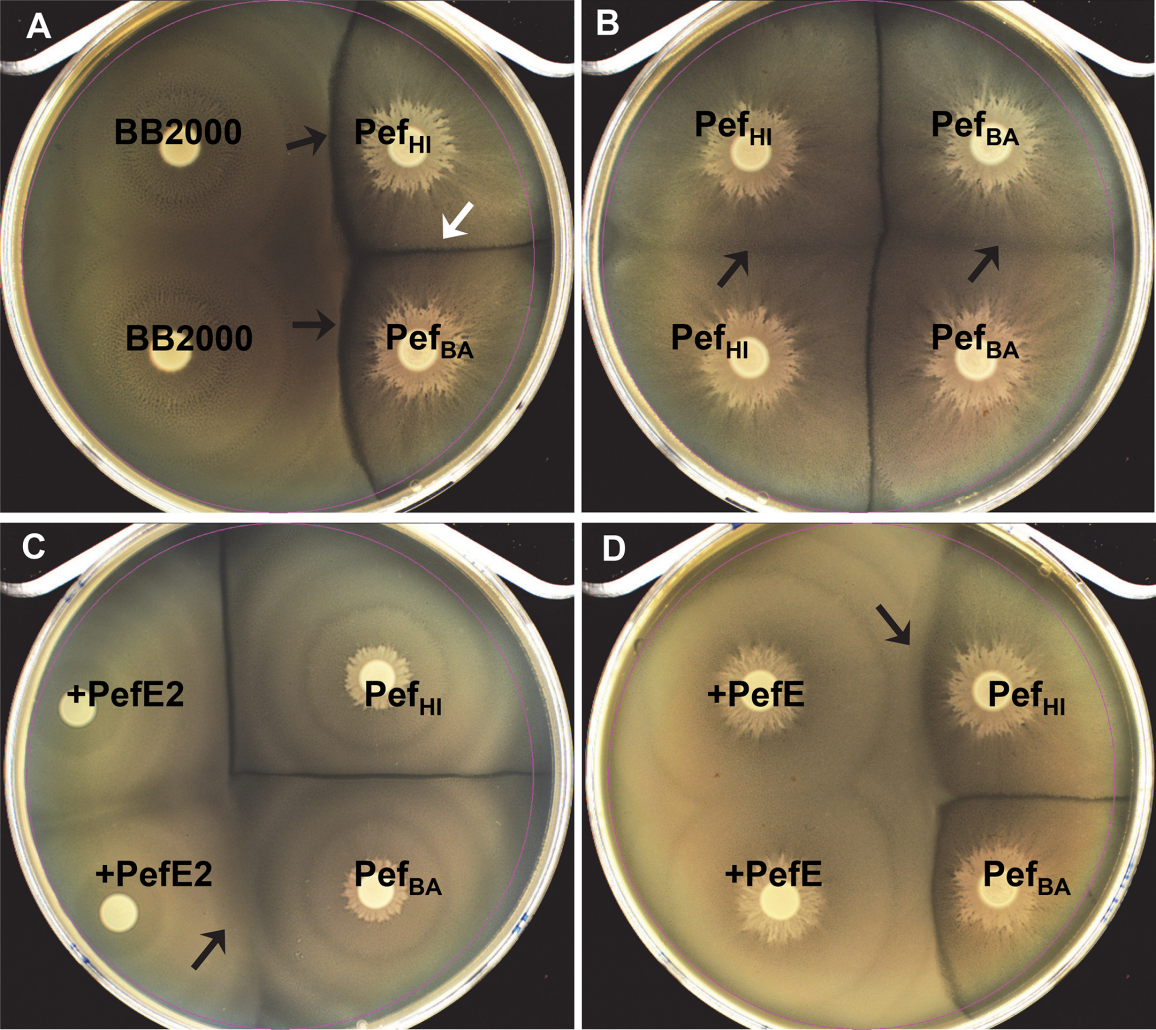
The *pef* operon is sufficient to create new Dienes-types in *P*. *mirabilis* BB2000 and immunity can be conferred by the cognate PefE immunity protein. **(A)** Introduction of the entire HI4320 *pef* operon (Pef_HI_) or the BA6163 *pef* operon (Pef_BA_) into isolate BB2000, which lacks a *pef* operon, creates strains that form Dienes lines with parental BB2000 (black arrows). A Dienes line also forms between BB2000 containing the HI4320 *pef* operon and BB2000 containing the BA6163 *pef* operon (white arrow) because they encode different *pefE* alleles. **(B)** Dienes line formation is not observed between BB2000 containing the same *pef* operon (black arrows). **(C)** Expression of *pefE2* provided immunity for BB2000 against BB2000 containing the BA6163 *pef* operon (Pef_BA_) (black arrow). BB2000 containing *pefE2* is not immune to BB2000 containing the HI4320 *pef* operon (Pef_HI_). **(D)** BB2000 expressing *pefE* is immune to BB2000 containing the HI4320 *pef* operon (black arrow), while *pefE* does not provide immunity against BB2000 containing the BA6163 *pef* operon.

Due to the observation of Dienes line formation between 9C1 containing the *pef* operon from HI4320 and 9C1 containing the *pef* operon from BA6163 (Fig 4C, boxed area) we performed gain of function experiments using *P*. *mirabilis* strain BB2000, which lacks any *pef* homolog in its genome [23]. As expected, introduction of either the entire HI4320 *pef* operon or the BA6163 *pef* operon into isolate BB2000 created a strain that formed a Dienes line with the parental wild-type BB2000 (Fig 5A). We also observed Dienes line formation between BB2000 containing the HI4320 *pef* operon and BB2000 containing the BA6163 *pef* operon (Fig 5B), presumably due to the different *pefE* alleles each operon encodes. As expected, Dienes line formation was not observed between two merging BB2000 strains containing the same *pef* operon (Fig 5B, black arrows). These results demonstrate that introduction of a single ‘new’ T6SS effector operon into *P*. *mirabilis* is sufficient to create a new kin recognition group or Dienes type and also confirms the unique specificities of each of the *pef* operons from BA6163 and HI4320.

Additional gain of function experiments were carried out by introducing the genes encoding individual HI4320 immunity proteins, PefE or PefE2, into BB2000 to provide immunity against BB2000 strains containing the *pef* operon from either HI4320 or BA6163. As expected, expression of PefE2 provided immunity for BB2000 against BB2000 containing the BA6163 *pef* operon (Fig 5C), providing clues to the specificity-conferring residues in PefE. BB2000 containing PefE2 was not immune to BB2000 containing the HI4320 *pef* operon (Fig 5C). Furthermore, BB2000 expressing PefE was immune to BB2000 containing the HI4320 *pef* operon (Fig 5D), while PefE did not provide immunity against BB2000 containing the BA6163 *pef* operon (Fig 5D). These experiments verify that immunity from PefE or PefE2 is specific to the effector function encoded by the cognate operon. Also, these findings confirm that PefE2 of HI4320 does function as an immunity factor, since kin recognition was demonstrated against BB2000 containing the BA6163 *pef* operon.

### Localization of the PefE specificity sequence

**Fig 6:**
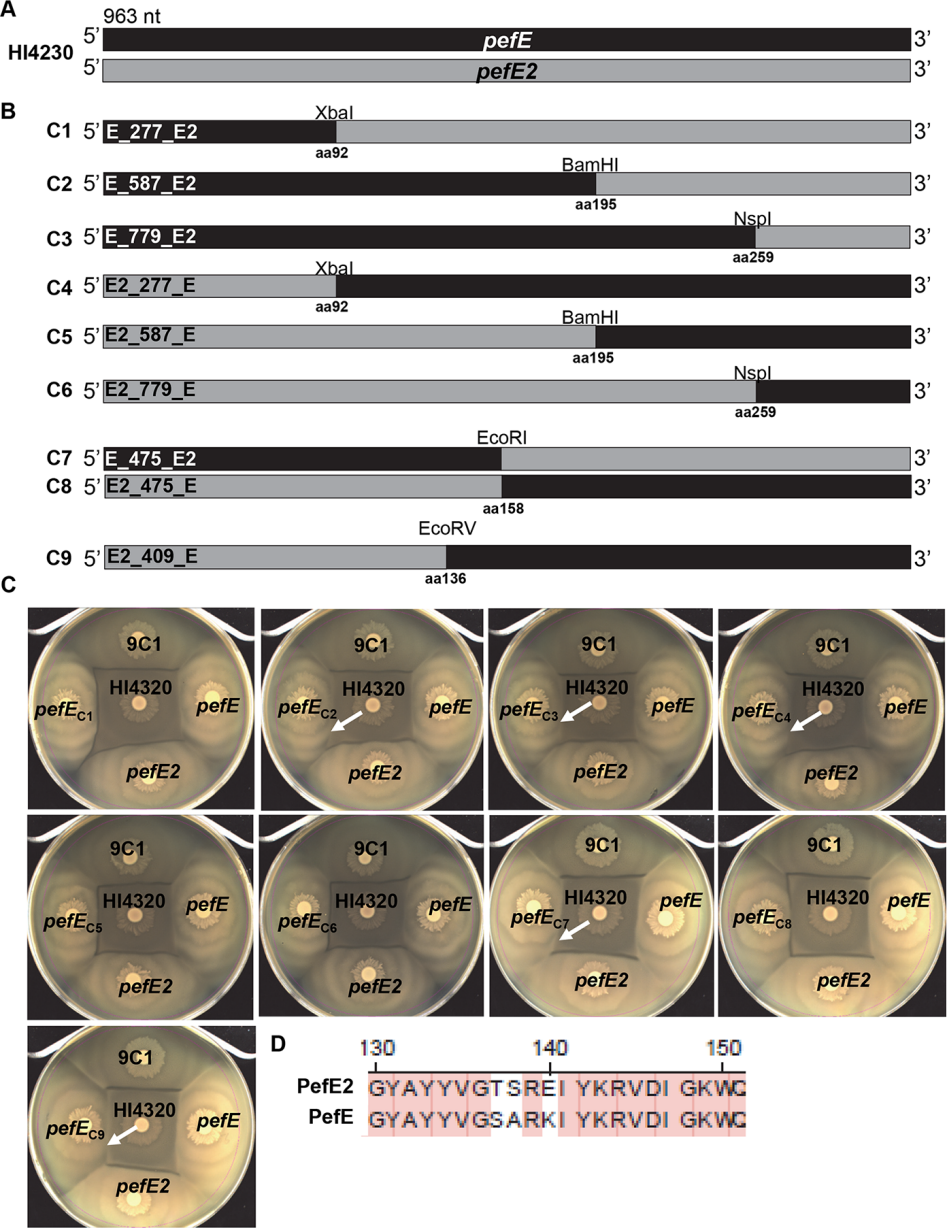
Chimeric immunity proteins constructed from PefE and PefE2 reveal amino acid residues responsible for specificity. **(A)** *P*. *mirabilis* HI4320 *pefE* (black) and *pefE2* (grey) were PCR-amplified to construct chimeric immunity proteins to determine regions within PefE and PefE2 responsible for immunity function. **(B)** Nine chimeras (C1-C9) composed of partial gene fragments from *pefE* and *pefE2* were created using conserved restriction sites (XbaI, BamHI, NspI, EcoRI, and EcoRV) located within the genes and ligated into pBAD-MycHisA. The amino acid sequence number of the 3’ end *pefE* or *pefE2* gene fragment is indicated below each restriction enzyme site and also depicts the point of fragment ligation. **(C)** Following induction by 10 mM L-arabinose, 9C1 expressing *pefE* restored immunity function against parent strain HI4320 (no Dienes line) in contrast to 9C1 containing pBAD empty vector or 9C1 expressing *pefE2*, which were unable to complement the immunity defect and thus result in formation of a Dienes line. Chimeric immunity proteins C2, C3, C4, C7, and C9 restored immunity against HI4320 (no Dienes line; white arrows). **(D)** Amino acid sequences of residues 130–150 from PefE and PefE2 are highly conserved except for residues 137, 138, and 140 suggesting that these residues are responsible for specificity of immunity.

To determine sites responsible for the specificity of immunity within the PefE protein, we constructed chimeras from the two distinct genes encoded by HI4320, *pefE* and *pefE2* (Fig 6A). Partial gene fragments of varying lengths were created using existing conserved restriction enzyme sites, allowing for the construction of nine unique full-length PefE chimeras, C1-C9 (Fig 6B). Each of these chimeras was first examined in mutant 9C1 to determine if the construct would complement the missing PefE and restore kin recognition with HI4320. In the Dienes line experiments, C2, C3, C4, C7, and C9 complemented the kin recognition defect of 9C1 against HI4320 and no Dienes line was observed (Fig 6C, white arrows), similar to the complementation observed by the positive control PefE in 9C1 against HI4320 (Fig 6C). Expression of C1, C5, C6, and C8 was unable to complement kin recognition in 9C1 and Dienes line formation was still observed against HI4320 (Fig 6C).

The chimeras were also tested in *P*. *mirabilis* BB2000 to confirm the immunity function of these chimeras against BB2000 containing the *pef* operon from HI4320. As expected no Dienes line was observed for C2, C3, and C4 when tested against BB2000 containing the *pef* operon from HI4320, suggesting that these chimeras contain a region of PefE that is required for immunity and kin recognition against BB2000 containing the HI4320 *pef* operon (S3 Fig). Dienes line formation was observed for BB2000 with C1, C5, and C6 tested against BB2000 containing the *pef* operon from HI4320 (S3 Fig). Taken together, these results show that the PefE sequence present in C2, C3, and C4 contains the determinant that encodes specificity for PefE immunity. By comparing C2, C3, and C4 and identifying the residues where the PefE sequences overlap we were able to determine that the region of specificity for PefE is located between aa92-195 (Fig 6B).

We observed that the remaining chimeras; C1, C5 and C6, have sequence homologous to PefE2 spanning residues aa92-195. Since immunity protein PefE2 is highly conserved to PefE from BA6163, we tested each of the chimeras for complementation of immunity against BB2000 containing the *pef* operon from BA6163. For these experiments, we again used strain BB2000 and tested each of the chimeras against BB2000 containing the BA6163 *pef* effector operon (S4 Fig). As expected, Dienes line formation was observed for all strains when *pefE* constructs were not induced. As observed previously, the Dienes line did not form between BB2000 containing the *pef* operon from BA6163 and BB2000 containing PefE2 from HI4320 following induction with 10 mM L-arabinose (black arrows) (S4 Fig). Only C1, C5, and C6 were able to provide kin recognition with BB2000 containing the BA6163 *pef* operon (white arrows), which resulted in the disappearance of the Dienes line (S4 Fig). In contrast, a Dienes line formed between BB2000 containing C2, C3, and C4 and BB2000 containing the *pef* BA6163 operon suggesting that these chimeras do not contain the specificity element for BA6163 PefE (S4 Fig) and again indicating the specificity determining region was located between aa92-195.

It was possible to further narrow down the specificity-encoding residues between aa92-195 by examining the results from C7-C9 that produce hybrid PefE and PefE2 proteins at residues aa158 and aa136 (Fig 6B). We found that C7 and C9 both complemented the recognition defect in mutant 9C1 lacking *pefE* (white arrows) (Fig 6C) while C8 was unable to complement 9C1 (Fig 6C). Because C7 and C9 PefE specific residues overlap between residues aa136-158 (Fig 6B), the kin recognition specificity determinant is likely encoded within this 22aa region. It is notable that PefE and PefE2 only diverge at three amino acids; aa137, 138, and 140, within this region (Fig 6D); thus, the information for kin identification may be encoded in as few as three amino acids within PefE.

All nine chimeras were also tested in a quantitative killing assay, since Dienes line formation results from lethal action of the T6SS [10]. In this assay, HI4320 killed the *pefE* mutant (9C1) over 7 orders of magnitude, and complementation with *pefE* restored survival (S5 Fig). As observed with the Dienes line kin recognition experiments, the chimeras that complemented 9C1 (*e*.*g*., the Dienes line is not seen between 9C1 with these chimeras and HI4320) exhibited less HI4320-mediated lethality/killing than the chimeras that were unable to complement in the Dienes line experiments. C2, C3, C4, C7, and C9 all reduced the level of killing by > 2 logs (S5 Fig), while C1, C5, C6, and C8 were indistinguishable from the vector control (pBAD) (S5 Fig). Regardless of whether a given chimera phenocopied PefE and complemented 9C1, all of the chimeric PefE proteins were functional (Table 2); for example C1, C5, and C6 phenocopy PefE2. Taken together, these chimera experiments provide proof that T6SS-mediated kin identification can be encoded by a specific region within a single gene, *pefE*.

**Table 2:** Immunity function and Dienes type for chimeric PefE:PefE2 proteins.

Construct	Description	Complements 9C1	Dienes line with *pef* operon HI4320	Dienes line with *pef* operon BA6163
**pefE**	**pefE**	**+**	**-**	**+**
**pefE2**	**pefE2**	**-**	**+**	**-**
**C1**	**E_277_E2**	**-**	**+**	**-**
**C2**	**E_587_E2**	**+**	**-**	**+**
**C3**	**E_779_E2**	**+**	**-**	**+**
**C4**	**E2_277_E**	**+**	**-**	**+**
**C5**	**E2_587_E**	**-**	**+**	**-**
**C6**	**E2_779_E**	**-**	**+**	**-**
**C7**	**E_475_E2**	**+**	**-**	**+**
**C8**	**E2_475_E**	**-**	**+**	**-**
**C9**	**E2_409_E**	**+**	**-**	**-**

### Modular organization of PefE variable regions

**Fig 7:**
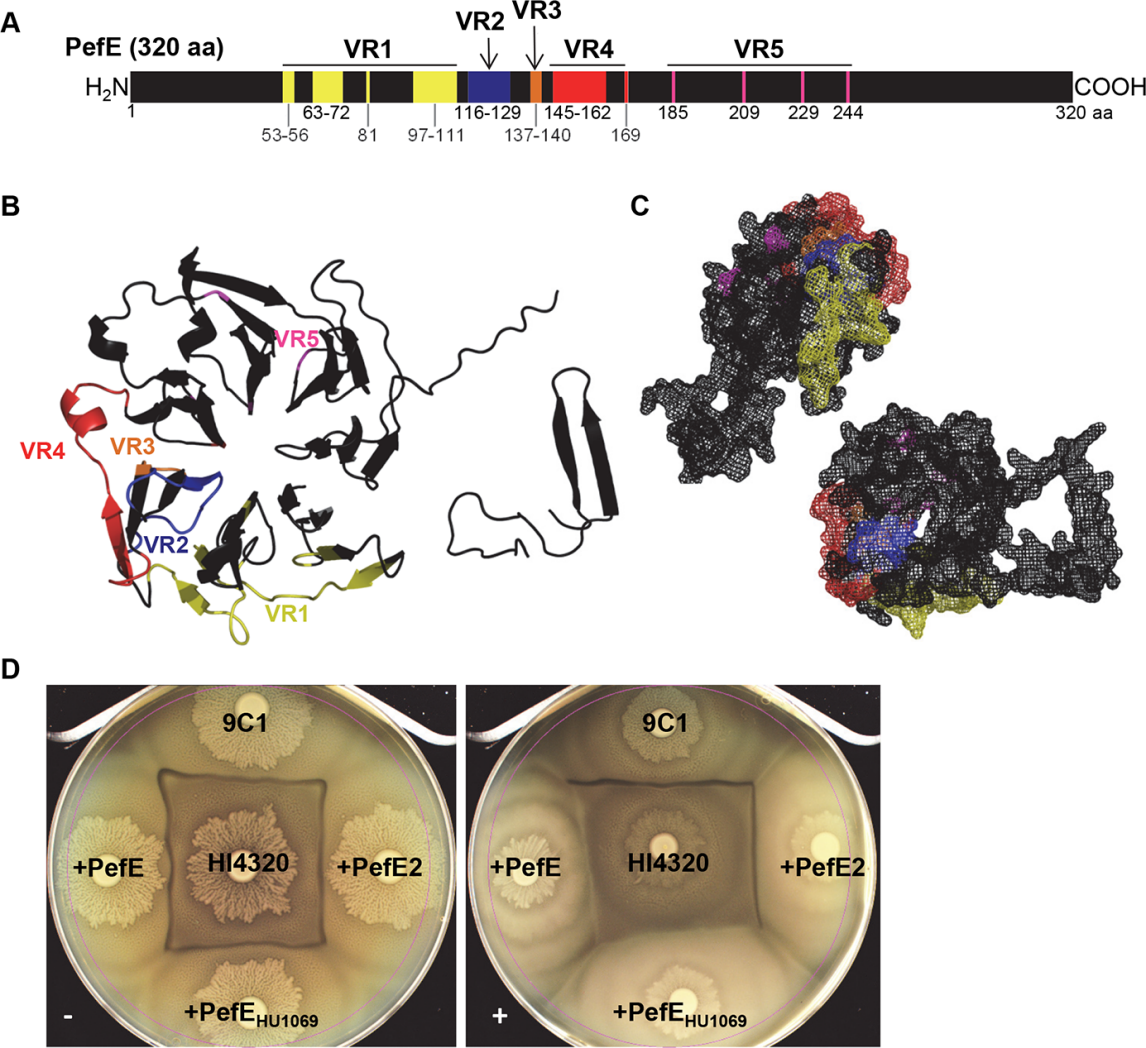
Alignment of PefE immunity proteins identifies five variable regions. **(A)** Alignment of the 320-amino acid PefE from 16 *P*. *mirabilis* isolates identified conserved regions (black) interspersed with 5 variable regions (VR1, VR2, VR3, VR4, VR5) that were less conserved. Within each variable region, 2–3 variants were identified. Assembly of these variant modules led to 7 versions of the PefE immunity protein among 16 clinical isolates. Three of these isolates encode 2 versions of PefE (HI4320, BA6163, PL105). **(B)** Phyre-predicted 3D structure of PefE is depicted as a ribbon diagram with the 5 variable regions superimposed and visualized by color. Variable regions predominate on one ‘side’ of the PefE model which adopts a beta-sheet rich bladed propeller fold. The colors in (B) correspond to the labeled variable regions from (A). **(C)** Space-filling model of PefE generated by PyMOL. The colors in (C) correspond to the labeled variable regions from (A and B). **(D)** Kin recognition is dependent on three amino acid residues from VR3. PefE from *P*. *mirabilis* isolate HU1069 was cloned and expressed in 9C1 (PefE_HU1069_) under non-inducing conditions (-) and induced with 10 mM L-arabinose (+).

Since minor PefE variants exhibit different specificities, we further investigated the sequences of *pefE* genes to determine if sequence diversity could be used to predict kin recognition between the strains. Genomic DNA isolated from 16 *P*. *mirabilis* clinical isolates was PCR-amplified using degenerate primers homologous to the three *pefE* genes of HI4320 and BA6163. Following sequence analysis, amino acid alignments were performed on all of the deduced PefE protein sequences from each *P*. *mirabilis* isolate. Like HI4320, two of the isolates, BA6163 and PL105, encoded two versions of PefE. Amino acid sequence alignments of all 19 PefE proteins depict conserved regions interspersed with five less-conserved variable regions called VR1, VR2, VR3, VR4, and VR5 (Fig 7A). Within three of these variable regions (VR1, VR4 and VR5), only two to three variants (termed V1, V2, or V3) were identified based on sequence identity. Upon assembly of these variant modules, seven versions of the PefE immunity protein were identified among 16 clinical isolates. The discussed kin recognition specificity element we identified using the chimeras is predicted to reside in VR3 (Fig 7A).

Using Phyre software [24], a predicted 3D structure of PefE was generated as a ribbon diagram and shows a β-sheet bladed propeller fold (Fig 7B). Visualization of the five variable regions within PefE was created using PyMOL software (Fig 7B) as were space-filling models of the predicted protein structure (Fig 7C). Interestingly, all five of the variable regions were superimposed onto one “side” of the putative PefE model (Fig 7B and 7C). Further, the specificity element VR3 appears to occupy a region closer to the center of the propeller relative to the other variable regions within the predicted structure (Fig 7B).

To determine if the identified specificity determinant encoded within VR3 plays a role in immunity, we cloned the *pefE* gene from isolate HU1069, and tested it for immune function. Isolate HU1069 PefE, like HI4320 PefE2, is divergent from HI4320 PefE at VR1 and VR5, but unlike PefE2, PefE from HU1069 encodes the same residues as PefE from HI4320 at the critical VR3 specificity element (S6 Fig). We reasoned that PefE from HU1069 would complement 9C1 because it contains the necessary kin recognition element. Indeed, 9C1 containing the *pefE* cloned from isolate HU1069 was recognized as kin by parental HI4320 as evidenced by the lack of a Dienes line following induction (Fig 7D). These data further support that the short run of amino acid residues encoded within VR3 are responsible for kin recognition.

### Site-directed mutagenesis of the PefE specificity element

Based on the results from chimeric PefE proteins and the *pefE* cloned from isolate HU1069 we reasoned that it might be possible to change an immunity protein that functions as PefE to one that functions as PefE2 by solely changing residues within the specificity element. Since only three amino acid residues are divergent between PefE and PefE2 within VR3, we constructed the following PefE substitutions at aa137, 138, and 140 with the amino acid residue found in PefE2: PefE_S137T, PefE_A138S, and PefE_K140E (Table 3). In addition to these three single point mutations, double site-directed mutants of all configurations as well as a triple site-directed mutant was created: PefE_S137T A138S, PefE_S137T K140E, PefE_A138S K140E, and PefE_S137T A138S K140E. Additional single and double site-directed mutants of PefE at residues aa137S and aa140K were constructed to introduce an alanine (A); PefE_S137A, PefE_K140A, and PefE_S137A K140A (Table 3). Surprisingly, all of the site-directed mutations where residues from PefE2 were substituted into PefE were unable to change the specificity; all constructs maintained function as PefE and none functioned as PefE2 (Table 3). However, by introducing alanines into the residues within the specificity element it was possible to disrupt immunity function entirely (Table 3); PefE S137A K140A double mutant was unable to function as PefE and could not complement 9C1 (S7 Fig, panel A)

**Table 3:** Immunity function and Dienes type for site-directed PefE mutants.

	Immunity against *pef* operon from HI4320	Immunity against *pef* operon from BA6163
**PefE**	**Y**	**N**
**PefE2**	**N**	**Y**
**E S137T**	**Y**	**N**
**E A138S**	**Y**	**N**
**E K140E**	**Y**	**N**
**E S137T A138S**	**Y**	**N**
**E S137T K140E**	**Y**	**N**
**E S137T A138S K140E**	**Y**	**N**
**E S137A**	**Y**	**N**
**E K140A**	**Y**	**N**
**E S137A K140A**	**N**	**N**
**E_475_E2**	**Y**	**N**
**E_475_E2 S137T**	**Y**	**N**
**E_475_E2 A138S**	**Y**	**N**
**E_475_E2 K140E**	**Y**	**N**
**E_475_E2 S137T A138S**	**Y**	**N**
**E_475_E2 S137T K140E**	**Y**	**Y**
**E_475_E2 A138S K140E**	**Y**	**Y**
**E_475_E2 S137T A138S K140E**	**Y**	**Y**

Because it was not possible to switch PefE to PefE2 function by exclusively changing the three divergent amino acids within the specificity sequence, the regions flanking the specificity element may also play a role in their ability to function as either PefE or PefE2. To test this possibility, we created the same site-directed mutations as described above within the chimeric PefE, C7 (E_475_E2), which functions as PefE (S7 Fig, panel C and D). C7 contains the N-terminus of PefE and includes the three PefE specific residues within the specificity element. While none of the site-directed mutations in C7 lost function as PefE, we found that changing residue 140 from K to E in combination with any other change from an PefE to a PefE2 residue in this region was sufficient for the construct to function as both PefE and PefE2 (Table 3). The E_475_E2 chimera containing K140E and either S137T or A138S or both all now provided immunity against the *pef* operon from BA6163 in addition to maintaining function as PefE and complementing 9C1 (Table 3).

## Discussion

Swarming in *P*. *mirabilis* is a multicellular behavior whereby cooperation provides the advantage of rapid locomotion for a population across a solid surface [25–27]. Kin discrimination is one mechanism to identify organisms that are most likely to contain traits that enable cooperative behavior [28, 29]. In bacteria, kin discrimination during cooperative swarming has been shown to occur in *P*. *mirabilis*, *Bacillus subtilis*, and *Myxococcus xanthus* [12–14, 30]. In *P*. *mirabilis*, genes involved in self-recognition have been identified [20]. The mechanism explaining self-recognition or the Dienes phenomenon in *P*. *mirabilis* has been shown to be dependent on the T6SS [10]. Indeed, when two non-identical isolates both have T6SS-encoded genes disrupted, the ability to recognize the opposing swarm population as non-kin is lost [10]. Thus, in *P*. *mirabilis* the Dienes phenomenon is wholly dependent on the T6SS and the ability to recognize kin results from being immune against the activity of T6SS effectors [10]. *P*. *mirabilis* isolates contain multiple T6SS effector operons [10] and it is unlikely that any given isolate will share the exact same complement of T6SS effectors, resulting in the ability to discriminate non-kin rather than identify kin. For example, strain HI4320 has five separate T6SS effector operons; strain BB2000 has three distinct effector operons, of these 8 effector operons, one is shared. The shared effector operons are allelic variants that do not provide cross-immunity (*ids* operon) [10, 20]. Therefore, kin discrimination between HI4320 and BB2000 is the result of the combined activity of 8 distinct effector operons.

Our studies of a T6SS effector operon have led to the conclusion that, in addition to a diverse arsenal of T6SS effector operons, subtle variation within a conserved effector operon contributes to kin recognition. In support of this idea, we have demonstrated that introduction of a *pef* effector operon into a *P*. *mirabilis* strain lacking a native *pef* operon creates a strain that no longer recognizes its parent strain as kin. Furthermore, when two otherwise isogenic strains harbor distinct *pef* operon allelic variants, both strains fail to recognize the parent as kin nor do they recognize one another as kin. We have demonstrated that this failure of two *pef* operon variants to recognize one another as kin results from the fact that the PefD- and PefE-encoding genes between the two operons are divergent. Taken together, these data show that T6SS-mediated killing and immunity can involve proteins that differ subtly between strains in addition to different sets of effector-immunity pairs. Furthermore, a toxic effector requires additional gene products encoded within the cognate operon to exert its lethal effects, and thus the *pef* T6SS effector operon is a first example of a non-canonical effector operon that goes beyond a two gene toxin-immunity encoding operon.

We reasoned that non-conserved amino acid residues within PefE should be responsible for the specificity of immunity (kin recognition). Through the PefE-PefE2 chimera experiments, we delineated a specific region responsible for kin identification to amino acids 136–158. Every chimera functioned identically to either PefE or PefE2 with respect to kin identification. We were further able to delineate this region because PefE and PefE2 diverge by only 3 amino acids in this variable region (VR3). We also showed that a *pefE* allele from isolate HU1069, which is identical to HI4320 in the VR3 region but divergent in other variable regions, was able to restore kin recognition to the HI4320 *pefE* mutant strain 9C1. Furthermore, loss of allele-specific immunity can be achieved by amino acid changes in a chimeric PefE protein. The data summarized in Table 3 show that E_475_E2 with amino acid change K140E and either S137T or A138S or both results in a PefE variant that can provide immunity against both *pef* operon alleles (HI4320 and BA6163). The result demonstrating a promiscuous immunity protein has important implications regarding the evolution of kin discrimination via the T6SS and suggests that new recognition types can occur via an intermediate immunity variant that would maintain protection in the absence of changes to a toxic effector protein.

The present study provides defining information regarding how variation within a single T6SS effector operon and adds to our understanding of how kin discrimination occurs in *P*. *mirabilis*. Subtle variation within a single T6SS effector operon allows for isolates to discriminate even between isolates that otherwise share identical effector operons. Indeed, we demonstrated that a single isolate encoding multiple effector operons could discriminate between self and non-self on the basis of *pefE* and *pefD* allelic variation alone. It is possible that kin recognition in *P*. *mirabilis* via the T6SS has evolved to maximize their cooperative swarming behavior. *P*. *mirabilis* populations can also eliminate cheaters by deploying the T6SS during swarming. Therefore, the T6SS can be considered a contact-dependent kin discriminatory mechanism that benefits multicellular cooperation. With regard to kin recognition, it is reasonable to conclude that the recipient cell is responsible for kin identification because recognition is survival and therefore, as we have shown, selective pressure occurs largely on the T6SS immunity gene.

## Methods and materials

### Bacterial strains and constructs

Strains and plasmids used in this study are listed in Table 1. *P*. *mirabilis* HI4320 was cultured from the urine of a nursing home resident with catheter-associated bacteriuria [31]. To observe Dienes line formation on swarm agar [lysogeny broth (LB) medium containing NaCl (10 g/L) and 1.5% agar] overnight bacterial cultures were spotted (5μl) and incubated at 37°C for 18 h. To test Dienes line complementation in the HI4320 9C1 mutant, *pefE2* was PCR-amplified from HI4320 and cloned under the control of the arabinose-inducible promoter in pBAD-MycHisA (Invitrogen). A *pefE* homolog in *P*. *mirabilis* clinical isolate BA6163 and HU1069 [32] was PCR-amplified and sequenced using primers homologous to *pefE* and *pefE2* of HI4320, respectively (University of Michigan Sequence Core, Applied Biosystems 3730xl DNA Analyzer). The complete BA6163 *pef* operon was PCR-amplified with Phusion High-Fidelity Polymerase (Thermo-Fisher Scientific), digested with restriction enzymes SphI and NotI (New England Biolabs), and ligated to the lineraized pGEN-MCS vector. Ampicillin or Rifampicin (100 μg/ml) and kanamycin (25 μg/ml) was added as necessary; to induce pBAD and pETite constructs a final concentration of 10 mM L-arabinose and 0.1 mM IPTG, respectively, was added to swarm agar; for pETite expression in LB medium a final concentration of 1 mM IPTG was used; for pETite repression glucose (0.5%) was added to overnight cultures.

### PefE gene amplification and sequence alignment

Genomic DNA of sixteen *P*. *mirabilis* clinical isolates [32] was examined for the presence *pefE*-like genes using three pairs of degenerate primers based on the *pefE* sequence of HI4320 and BA6163, and the *pefE2* sequence of HI4320. PCR products were sequenced (University of Michigan Sequence Core, Applied Biosystems 3730xl DNA Analyzer), translated into amino acid sequences, and compared by multiple sequence alignment using Clustal W in MegAlign (DNASTAR).

### Bacterial killing assays

Bacterial killing assays were performed by mixing strains in a 1:1 ratio and spotted onto swarm agar (10 g NaCl/L). Following overnight incubation at 37°C, the entire swarm was collected, serially diluted, and plated onto LB medium containing NaCl (0.5 g/L) and 1.5% agar with rifampicin for HI4320 (Rif^R^) or no antibiotic for rifampicin-sensitive *Proteus* clinical isolates to determine CFU/ml. The output value was compared to the input value to quantify HI4320’s susceptibility to killing by other clinical isolates. HI4320 killing is reported as [(CFU of test strain/CFU of HI4320)_output_]/[(CFU of test strain/CFU of HI4320)_input_].

### Chimera construction

Partial gene fragments of genomic DNA sequences, *pefE* and *pefE2*, of *P*. *mirabilis* HI4320 were PCR amplified with oligonucleotides containing restriction enzyme sites and EasyA. The restriction enzyme sites; XbaI, BamHI, and NspI located within *pefE* and *pefE2* were chosen based upon their proximities to divide the length of the sequences into thirds and their ability to only cut once within the conserved sequences. Three additional chimeras were constructed using unique restriction enzyme sites EcoRI and EcoRV. Oligonucleotides and respective sequences used in these experiments are listed in (S1 Table). Digested PCR products of the *pefE* or *pefE2* fragments were ligated into linearized pGEN-MCS previously double-digested with NcoI and KpnI and CIP treated to dephosphorylate the 5’ and 3’ ends. Clones were screened by PCR amplification for the expected size insert within the multiple cloning site of pGEN and confirmed by sequence analysis. Chimeras used in this study are identified as C1, C2, C3, C4, C5, C6, C7, C8, and C9 and were also given a unique identifier based on sequence. These unique identifiers were generated based upon the 5’ partial gene fragment (either E for *pefE* or E2 for *pefE2*) followed by the nucleotide number where it is joined to the reciprocal gene fragment (either E for *pefE* or E2 for *pefE2*) at the 3’ end. Chimeras were transformed into electrocompetent cells of *P*. *mirabilis* HI4320 immunity mutant 9C1 and wild-type *P*. *mirabilis* BB2000 for further analysis in immunity complementation experiments.

### Site-Directed Mutagenesis

Desired point mutations within PefE were constructed using the Phusion Site-Directed Mutagenesis Kit (Thermo Fisher). Briefly, target plasmid pBAD-PefE-MycHisA was PCR amplified with two outward facing 5'-phosphorylated primers. Point mutations were introduced by one or more mismatches in the forward binding mutagenic primer. PefE point mutations at aa 137, 138, and 140 were created and substituted with the amino acid residue found in PefE2; PefE_aa137 serine (S) to threonine (T), PefE_aa138 alanine (A) to serine (S), PefE_aa140 lysine (K) to glutamic acid (E). In addition to these three single point mutations, double site-directed mutants of all configurations as well as a triple site-directed mutant was created; PefE_S137T A138S, PefE_S137T K140E, PefE_A138S K140E, and PefE_S137T A138S K140E. Additional single and double site-directed mutants of PefE at residues aa137S and aa140K were constructed to introduce an alanine (A); PefE_S137A, PefE_K140A, and PefE_S137A K140A. Oligonucleotides and respective sequences used in these experiments are listed in (S1 Table). Following PCR amplification, parental plasmid DNA was digested with DpnI for two hours at 37°C. Rapid ligation of the PCR products was performed with T4 DNA ligase (Thermo Fisher). The resulting circularized plasmid was N-butanol precipitated and the pellet was transformed into electrocompetent Top 10 *E*. *coli* (Life Technologies). Transformants were screened by colony PCR with primers amplifying across the pBAD-MycHisA multiple cloning site containing PefE. Clones with a correct product size of 1 Kb were verified by sequence analysis to confirm each point mutation (University of Michigan Sequence Core, Applied Biosystems 3730xl DNA Analyzer). Correct site-directed PefE plasmids were transformed into *P*. *mirabilis* HI4320 immunity mutant 9C1 and *P*. *mirabilis* BB2000 for immunity complementation experiments.

### PefD toxicity

To determine whether PefD in *P*. *mirabilis* HI4320 is the toxic effector of the *pef* operon, the entire *pefD* sequence and the tox-only domain of *pefD* was PCR amplified and ligated into the pETite N-His vector which has a 6xHis affinity tag fused to the amino terminus (Lucigen Expresso-T7 Cloning and Expression system). The resulting constructs were transformed into HI control 10G cells by heat shock and transformants were selected on kanamycin agar plates. HI control 10G cells harbor a single copy BAC plasmid carrying an engineered *lacI*^q1^ repressor allele responsible for tightly regulating and minimizing background transcription of the pETite plasmid which is optimal for construction with toxic proteins. Transformants were screened by colony PCR for the correct product size of 1.5 Kb (*pefD*) or 300 bp (Tox-only domain) and verified by sequence analysis (University of Michigan Sequence Core, Applied Biosystems 3730xl DNA Analyzer). For protein expression the correct pETite N-His-PefD and pETite N-His-Tox-PefD construct was transformed into HI control BL21 (DE3) cells (Lucigen) and induced in LB medium with 1 mM IPTG. Expression of pETite N-His-PefD and pETite N-His-Tox-PefD was verified by Western blot using THE His Tag HRP antibody (GenScript). Toxicity experiments of pETite N-His-PefD and pETite N-His-Tox-PefD were performed on swarm agar containing 0.1 mM IPTG.

### Ethics statement

The wild-type bacterial isolates used in this study were collected in Baltimore, Maryland in a prior study [32]. All isolates were collected with consent and were anonymized.

## Supporting information

S1 Table Primers and oligonucleotides. (XLSX)

S1 Fig Expression of PefD or the predicted nuclease domain from PefD inhibits growth of *E*. *coli*. Positive control protein (PC), PefD, or the nuclease domain (Tox) were expressed in *E*. *coli* BL21 in LB medium with kanamycin (closed symbols) and with kanamycin and 1 mM IPTG (open symbols) to induce protein production. Optical density at 600 nm was monitored for 20 hours. All strains induced with IPTG exhibited a growth delay and those expressing PefD or Tox exhibited greatest growth defect and reached a lower final density.
(TIF)

S2 Fig Expression of PefE does not reverse the growth defect in *E*. *coli* expressing PefD or the predicted nuclease domain of PefD. PefE and PefD or PefE and the nuclease domain (Tox) were expressed in *E*. *coli* BL21 in LB medium with kanamycin and ampicillin (open symbols) and with kanamycin, ampicillin and 1 mM IPTG to induce PefD or Tox (closed symbols) and with kanamycin, ampicillin, 1 mM IPTG to induce PefD or Tox, and 10 mM L-arabinose to induce PefE (closed symbols). Optical density at 600 nm was monitored for 20 hours. All strains induced with IPTG exhibited a growth delay and those induced with L-arabinose to express PefE did not restore growth.
(TIF)

S3 Fig Specificity of PefE immunity is encoded in regions of chimera 2, 3, and 4. Following induction on 10 mM L-arabinose (+), *pefE* cloned from HI4320 and expressed in BB2000 restored immunity against BB2000 containing the same immunity gene, *pefE* (black arrow). BB2000 containing pBAD empty vector alone or containing *pefE2* cloned from HI4320 were unable to restore immunity against parent strain BB2000 expressing *pefE* cloned from strain HI4320. Expression of chimera 1, 5, or 6 (PefE_C1_, PefE_C5_, PefE_C6_) in BB2000 did not restore immunity against BB2000 containing *pefE* cloned from HI4320; however, chimera 2, 3, and 4 (PefE_C2_, PefE_C3_, PefE_C4_) expressed in BB2000 restored immunity against BB2000 containing *pefE* cloned from strain HI4320 (white arrows).
(TIF)

S4 Fig Specificity of PefE2 immunity is encoded in amino acid residues that are in common in chimeras 1, 5, and 6. Following induction on 10 mM L-arabinose, Dienes line formation was observed between BB2000 expressing the *pef* operon from BA6163 (Pef_BA_) and BB2000 containing pBAD empty vector and BB2000 expressing *pefE*. BB2000 expressing Pef_BA_ is immune to BB2000 expressing *pefE2* cloned from HI4320 (black arrows). Expression of chimera 2, 3, or 4 (PefE_C2_, PefE_C3_, PefE_C4_) in BB2000 did not restore immunity against BB2000 expressing Pef_BA_; however, chimera 1, 5, and 6 (PefE_C1_, PefE_C5_, PefE_C6_) expressed in BB2000 restored immunity against BB2000 expressing Pef_BA_ (white arrows).
(TIF)

S5 Fig PefE chimeras that restore kin recognition are less susceptible to killing. Killing assays of 9C1 against the parental HI4320 are reported as [(CFU of 9C1 strain/CFU of HI4320)output] / [(CFU of 9C1 strain/CFU of HI4320)input]. Strain HI4320 kills mutant 9C1 by 7-logs (pBAD). Mutant 9C1 complemented with PefE is outcompeted by 2-logs. 9C1 containing C2-C4, C7, and C9 all merge with HI4320 and do not form a Dienes line.
(TIF)

S6 Fig PefE from HI4320 and HU1069 share the same critical VR3 residues. Alignment of HI4320 PefE and HU1069 PefE predicted amino acid sequences. Amino acids boxed in red indicate the matching residues at VR3. PefE from HU1069 restores kin recognition when introduced into HI4320 *pefE* mutant 9C1.
(TIF)

S7 Fig Two of three residues within the specificity region of PefE are required for immunity function. **(A)** *P*. *mirabilis* HI4320 forms a Dienes line with immunity mutant 9C1 (pBAD) and immunity is restored by expression of PefE (white arrow). Alanine substitution at residues 137 and 140 (S137A K140A) disrupt PefE immunity function and a Dienes line forms (black arrow). Site-directed mutagenesis switching PefE residues to PefE2 residues (S137T A138S K140E) partially disrupts immunity function as PefE and in **(B)** provides immunity function as PefE2 in BB2000 against BB2000 expressing the *pef* operon from BA6163. **(C)** and **(D)** Chimeric PefE C7 (E475E2) functions as PefE and not as PefE2. Site-directed mutation of C7 containing K140E and any additional change from E to E2 residue is sufficient to change function of C7 from PefE to PefE2 immunity phenotype (white arrows).
(TIF)
