# Supplementary figures and images for: Subtle variation within conserved effector operon gene products contributes to T6SS-mediated killing and immunity
Source: PLoS Pathog. 2017 Nov 20;13(11):e1006729. doi: 10.1371/journal.ppat.1006729 (PMC5714391; doi:10.1371/journal.ppat.1006729)

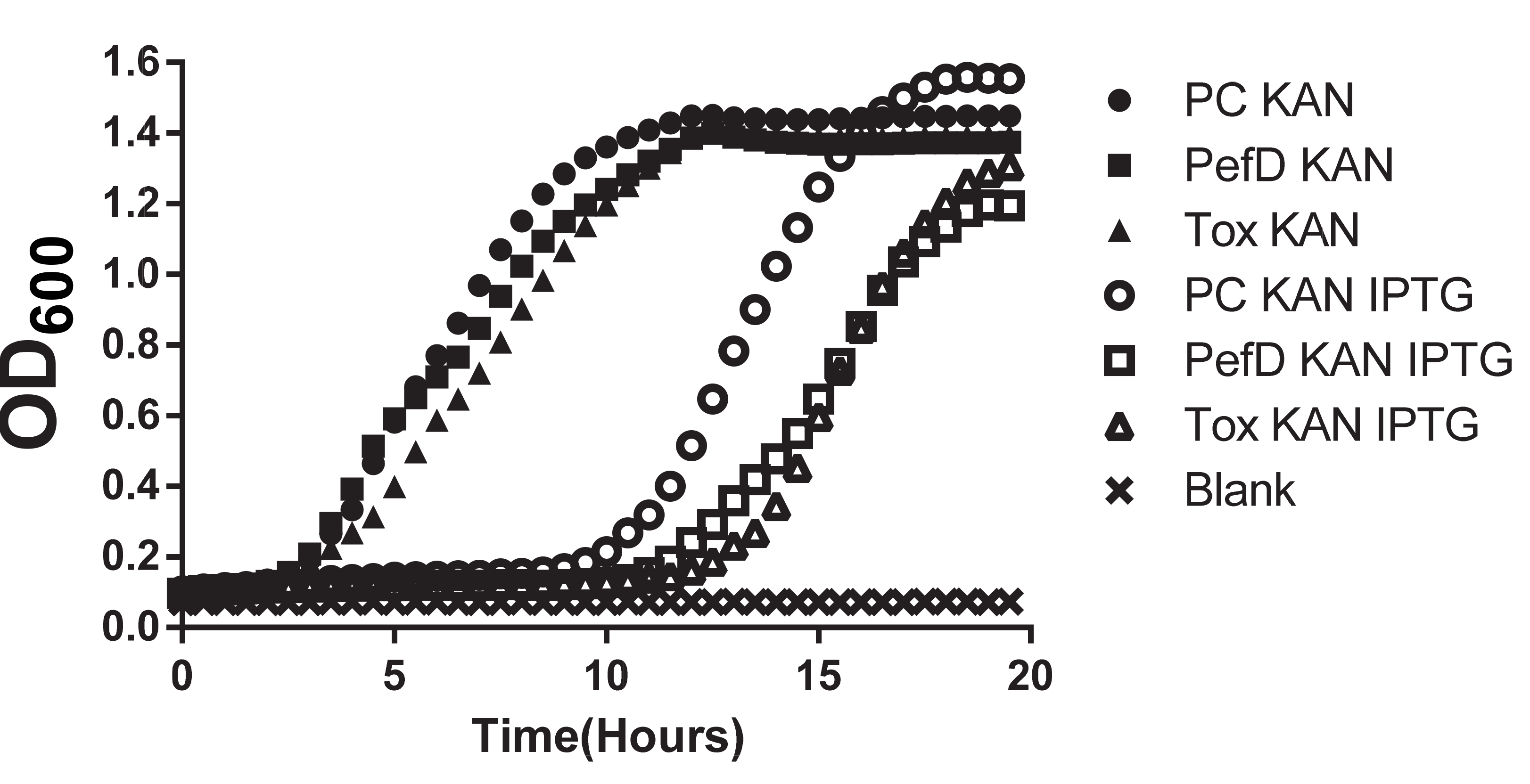

Supplement: S1 Fig — Positive control protein (PC), PefD, or the nuclease domain (Tox) were expressed in E. coli BL21 in LB medium with kanamycin (closed symbols) and with kanamycin and 1 mM IPTG (open symbols) to induce protein production. Optical density at 600 nm was monitored for 20 hours. All strains induced with IPTG exhibited a growth delay and those expressing PefD or Tox exhibited greatest growth defect and reached a lower final density. (TIF) [file ppat.1006729.s002.tif]

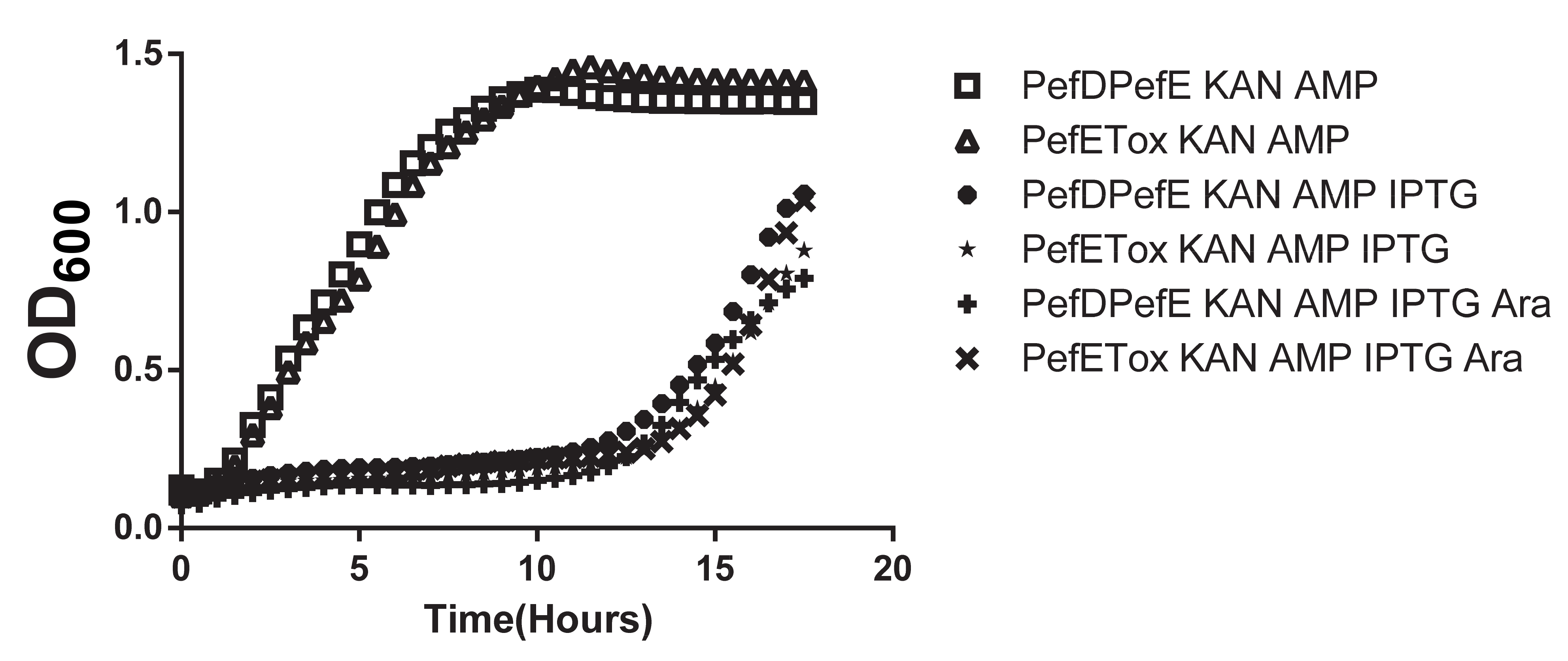

Supplement: S2 Fig — PefE and PefD or PefE and the nuclease domain (Tox) were expressed in E. coli BL21 in LB medium with kanamycin and ampicillin (open symbols) and with kanamycin, ampicillin and 1 mM IPTG to induce PefD or Tox (closed symbols) and with kanamycin, ampicillin, 1 mM IPTG to induce PefD or Tox, and 10 mM L-arabinose to induce PefE (closed symbols). Optical density at 600 nm was monitored for 20 hours. All strains induced with IPTG exhibited a growth delay and those induced with L-arabinose to express PefE did not restore growth. (TIF) [file ppat.1006729.s003.tif]

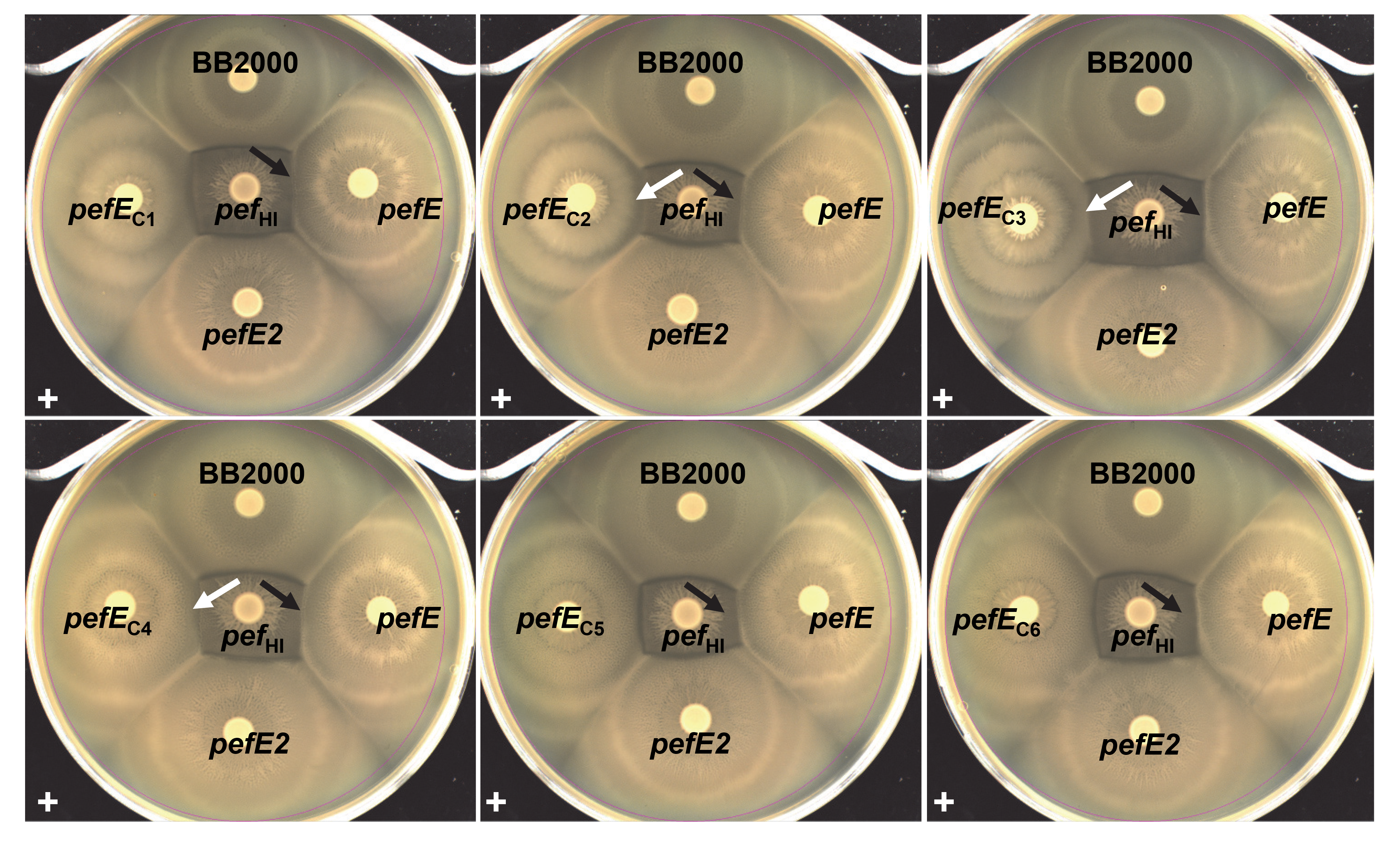

Supplement: S3 Fig — Following induction on 10 mM L-arabinose (+), pefE cloned from HI4320 and expressed in BB2000 restored immunity against BB2000 containing the same immunity gene, pefE (black arrow). BB2000 containing pBAD empty vector alone or containing pefE2 cloned from HI4320 were unable to restore immunity against parent strain BB2000 expressing pefE cloned from strain HI4320. Expression of chimera 1, 5, or 6 (PefEC1, PefEC5, PefEC6) in BB2000 did not restore immunity against BB2000 containing pefE cloned from HI4320; however, chimera 2, 3, and 4 (PefEC2, PefEC3, PefEC4) expressed in BB2000 restored immunity against BB2000 containing pefE cloned from strain HI4320 (white arrows). (TIF) [file ppat.1006729.s004.tif]

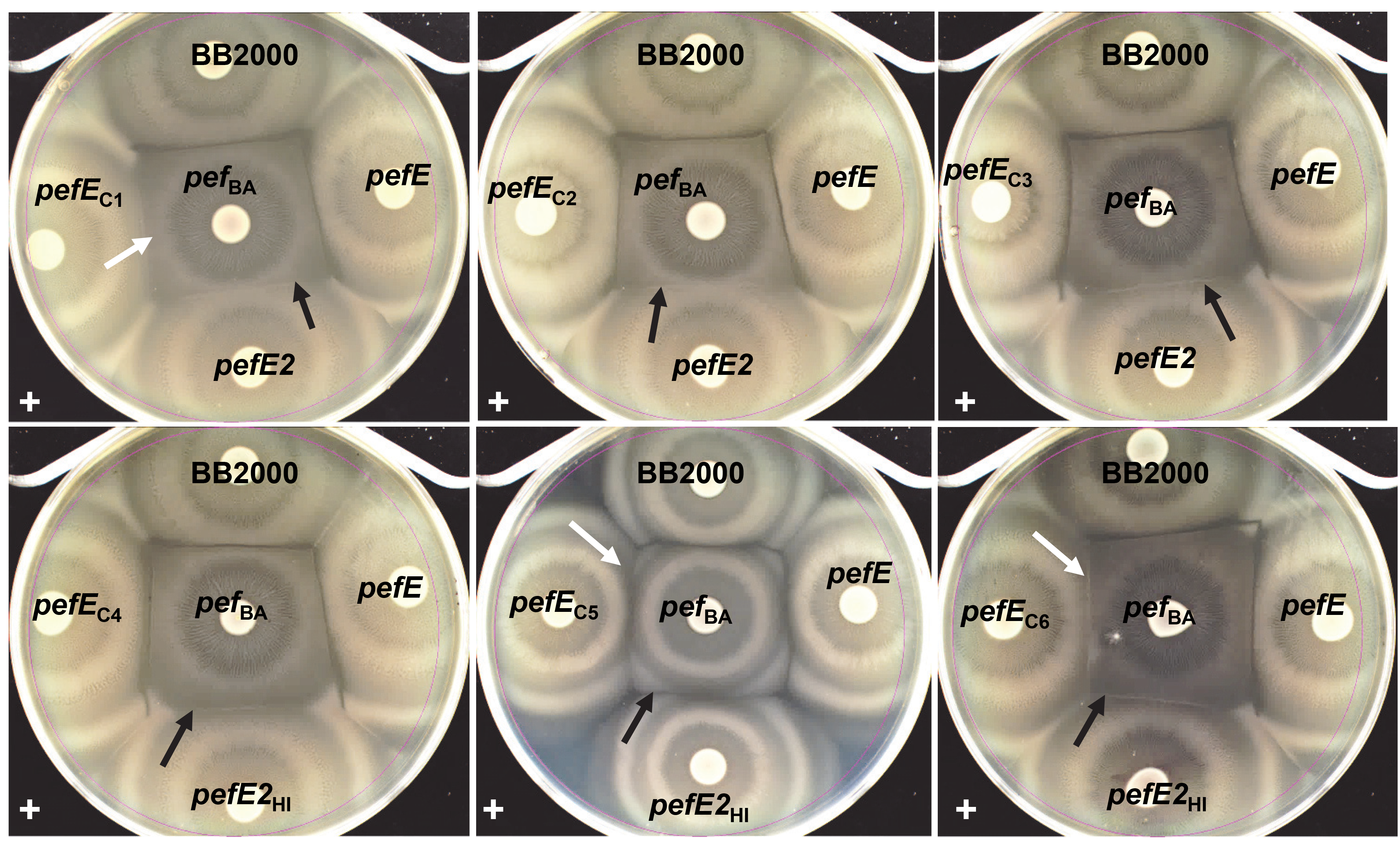

Supplement: S4 Fig — Following induction on 10 mM L-arabinose, Dienes line formation was observed between BB2000 expressing the pef operon from BA6163 (PefBA) and BB2000 containing pBAD empty vector and BB2000 expressing pefE. BB2000 expressing PefBA is immune to BB2000 expressing pefE2 cloned from HI4320 (black arrows). Expression of chimera 2, 3, or 4 (PefEC2, PefEC3, PefEC4) in BB2000 did not restore immunity against BB2000 expressing PefBA; however, chimera 1, 5, and 6 (PefEC1, PefEC5, PefEC6) expressed in BB2000 restored immunity against BB2000 expressing PefBA (white arrows). (TIF) [file ppat.1006729.s005.tif]

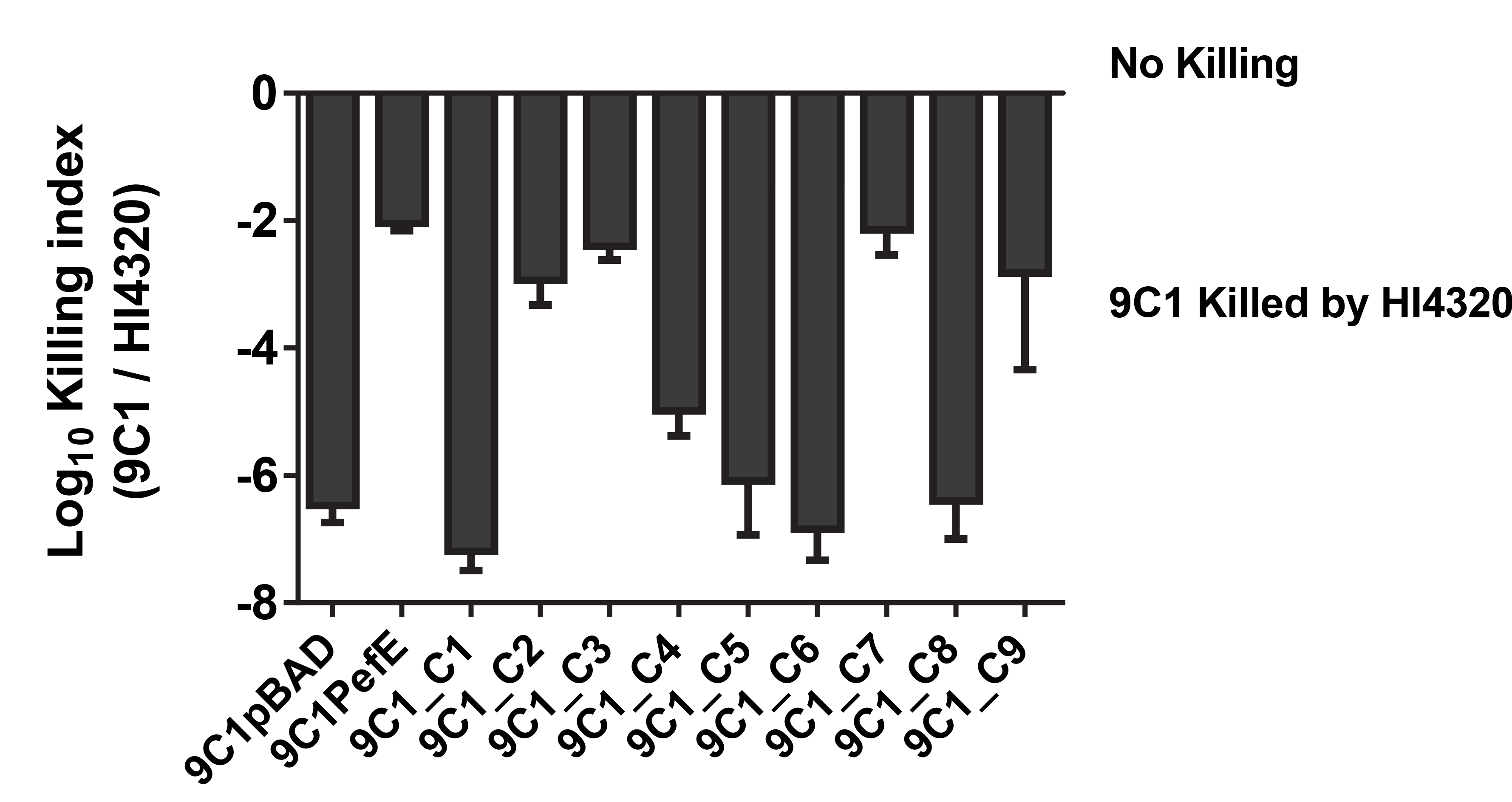

Supplement: S5 Fig — Killing assays of 9C1 against the parental HI4320 are reported as [(CFU of 9C1 strain/CFU of HI4320)output] / [(CFU of 9C1 strain/CFU of HI4320)input]. Strain HI4320 kills mutant 9C1 by 7-logs (pBAD). Mutant 9C1 complemented with PefE is outcompeted by 2-logs. 9C1 containing C2-C4, C7, and C9 all merge with HI4320 and do not form a Dienes line. (TIF) [file ppat.1006729.s006.tif]

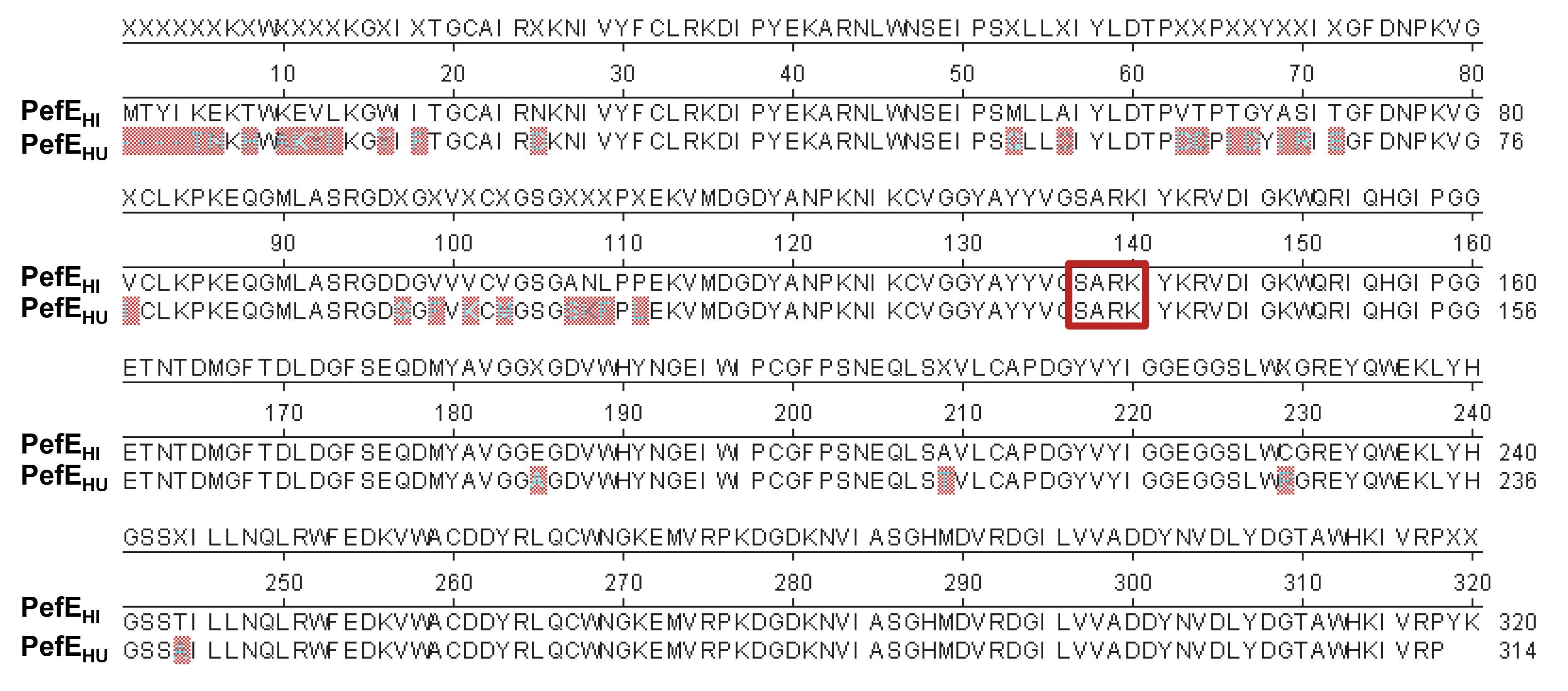

Supplement: S6 Fig — Alignment of HI4320 PefE and HU1069 PefE predicted amino acid sequences. Amino acids boxed in red indicate the matching residues at VR3. PefE from HU1069 restores kin recognition when introduced into HI4320 pefE mutant 9C1. (TIF) [file ppat.1006729.s007.tif]

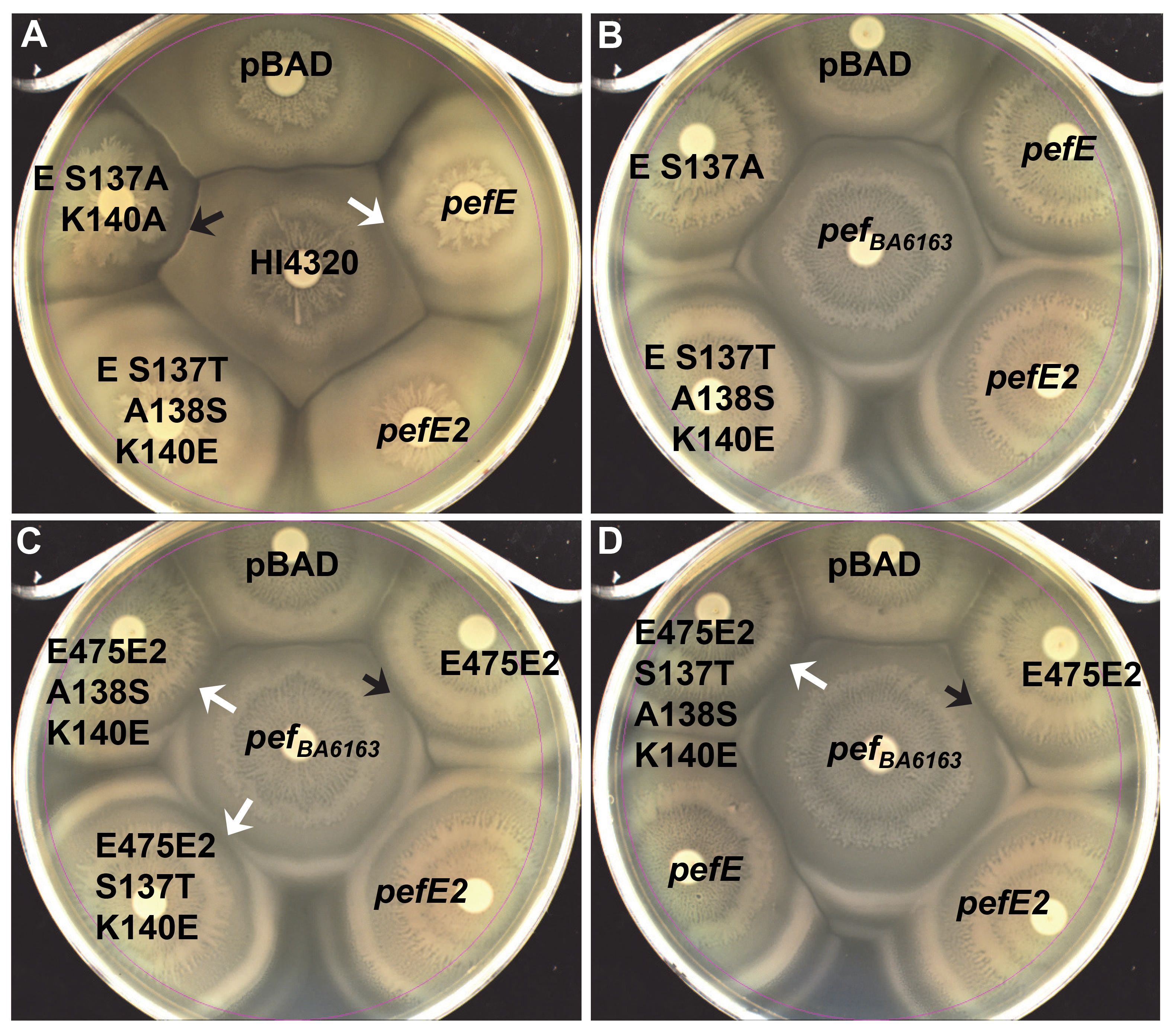

Supplement: S7 Fig — (A) P. mirabilis HI4320 forms a Dienes line with immunity mutant 9C1 (pBAD) and immunity is restored by expression of PefE (white arrow). Alanine substitution at residues 137 and 140 (S137A K140A) disrupt PefE immunity function and a Dienes line forms (black arrow). Site-directed mutagenesis switching PefE residues to PefE2 residues (S137T A138S K140E) partially disrupts immunity function as PefE and in (B) provides immunity function as PefE2 in BB2000 against BB2000 expressing the pef operon from BA6163. (C) and (D) Chimeric PefE C7 (E475E2) functions as PefE and not as PefE2. Site-directed mutation of C7 containing K140E and any additional change from E to E2 residue is sufficient to change function of C7 from PefE to PefE2 immunity phenotype (white arrows). (TIF) [file ppat.1006729.s008.tif]
